# Targeting MFAP5 in cancer-associated fibroblasts sensitizes pancreatic cancer to PD-L1-based immunochemotherapy via remodeling the matrix

**DOI:** 10.1038/s41388-023-02711-9

**Published:** 2023-05-08

**Authors:** Yi Duan, Xiaozhen Zhang, Honggang Ying, Jian Xu, Hanshen Yang, Kang Sun, Lihong He, Muchun Li, Yongtao Ji, Tingbo Liang, Xueli Bai

**Affiliations:** 1 Department of Hepatobiliary and Pancreatic Surgery, The First Affiliated Hospital, School of Medicine, Zhejiang University, Hangzhou, 310000 Zhejiang China; 2 Zhejiang Provincial Key Laboratory of Pancreatic Disease, The First Affiliated Hospital, School of Medicine, Zhejiang University, Hangzhou, 310000 Zhejiang China; 3 Zhejiang Provincial Innovation Center for The Study of Pancreatic Diseases, Zhejiang University, Hangzhou, 310000 Zhejiang China; 4 Zhejiang Provincial Clinical Research Center for The Study of Hepatobiliary & Pancreatic Diseases, Zhejiang University, Hangzhou, 310000 China; 5 Cancer Center, Zhejiang University, Hangzhou, 310000 China; 6 Research Center for Healthcare Data Science, Zhejiang Lab, Hangzhou, 310000 Zhejiang China

**Keywords:** Cancer microenvironment, Tumour immunology, Acetylation

## Abstract

Highly desmoplastic and immunosuppressive tumor microenvironment (TME) in pancreatic ductal adenocarcinoma (PDAC) contributes to tumor progression and resistance to current therapies. Clues targeting the notorious stromal environment have offered hope for improving therapeutic response whereas the underlying mechanism remains unclear. Here, we find that prognostic microfibril associated protein 5 (MFAP5) is involved in activation of cancer-associated fibroblasts (CAFs). Inhibition of MFAP5^high^CAFs shows synergistic effect with gemcitabine-based chemotherapy and PD-L1-based immunotherapy. Mechanistically, MFAP5 deficiency in CAFs downregulates HAS2 and CXCL10 via MFAP5/RCN2/ERK/STAT1 axis, leading to angiogenesis, hyaluronic acid (HA) and collagens deposition reduction, cytotoxic T cells infiltration, and tumor cells apoptosis. Additionally, in vivo blockade of CXCL10 with AMG487 could partially reverse the pro-tumor effect from MFAP5 overexpression in CAFs and synergize with anti-PD-L1 antibody to enhance the immunotherapeutic effect. Therefore, targeting MFAP5^high^CAFs might be a potential adjuvant therapy to enhance the immunochemotherapy effect in PDAC via remodeling the desmoplastic and immunosuppressive microenvironment.

## Introduction

PDAC is among the most malignant solid tumors with an extremely poor 5-year survival [1]. In clinic, combined application of chemotherapy and immunotherapy has improved the clinical outcomes of PDAC patients [2, 3]. Although immune checkpoint blockade (ICB) monotherapies have produced promising results in clinical trials of certain cancers, such as non-small-cell lung cancer, clear cell renal cell carcinoma and melanoma, zero response is observed in clinical treatment of PDAC [3, 4]. Notably, PDAC is recognized as “cold tumor” or “immune desert” with low immune response, and tumor-infiltrating lymphocytes (TILs) are difficult to be recruited into the immunosuppressive tumor niches [5]. The tumor-induced privileged site created by surrounding CAFs and matrix components is one of the major causes for immune escape [6, 7]. Therefore, stroma-targeted therapies are of vital importance to reshape the dense desmoplastic stroma and provide structural foundation for re-sensitization of PDAC to the current ICB or chemotherapies.

The highly desmoplastic and immunosuppressive TME of PDAC largely contributes to the malignant tumor progression, chemotherapeutic resistance and low response to immunotherapies [8, 9]. Over the past few years, increasing studies have focused on stroma-targeted strategies to improve therapeutic effects and clinical outcomes of PDAC patients [10]. As a fundamental component of the compact stroma, CAFs play a central role in stroma construction and crosstalk with tumor cells via extracellular matrix (ECM), including collagens, HA, fibronectin, laminin as well as ranges of chemokines and cytokines [11–13]. CAFs were transformed from activated pancreatic stellate cells (PSC) and were identified with high expression of α-smooth muscle actin (α-SMA) protein [14]. Recently, increasing CAF-specific targets involved in tumor progression and immune regulation have been explored and have shown potent adjuvant effect in mouse models [15, 16]. However, the high tumoral heterogeneity, unclear activation mechanisms of CAFs and the complex regulatory networks in TME have brought uncertainty to actual clinical response [17, 18]. Of note, it is inadvisable to completely eradicate CAFs or HA, for it could reversely enhance malignant tumoral characteristics in clinic [19, 20]. Therefore, alternative strategies that can reshape the stromal matrix rather than eliminate the carcinogenic components are urgently required.

MFAP5 was reported overexpressed in CAFs in ovarian cancer, and was associated with malignant tumor progression and chemoresistance of ovarian cancer, prostate cancer and cholangiocarcinoma [21–25]. Additionally, MFAP5 was regarded as a specific ECM indicator in esophageal squamous cell carcinoma and was used to indicate one subset of CAFs in single cell sequencing analysis [26]. Besides, in breast cancer, single cell sequencing analysis revealed that MFAP5 indicating myofibroblastic CAFs was involved in ECM organization and wound-healing features [26]. However, immune-related roles and the underlying mechanisms of MFAP5 in PDAC are still unknown.

In the present study, primary CAFs derived from both human and mouse PDAC tumors were isolated and cultured. We found that prognostic MFAP5 was highly expressed in myofibroblasts and contributed to the formation of the physical desmoplastic barrier and immunosuppressive TME. Notably, MFAP5 deficiency remodels the matrix via inhibiting CAF activation and increases infiltration of cytotoxic CD8^+^ T cells to tumor niches. Moreover, inhibition of MFAP5^high^CAFs can synergize with PD-L1-based immunochemotherapy, indicating MFAP5 as a potential stromal target for PDAC treatment.

## Results

### Prognostic MFAP5 is mainly overexpressed in myofibroblasts

**Fig. 1:**
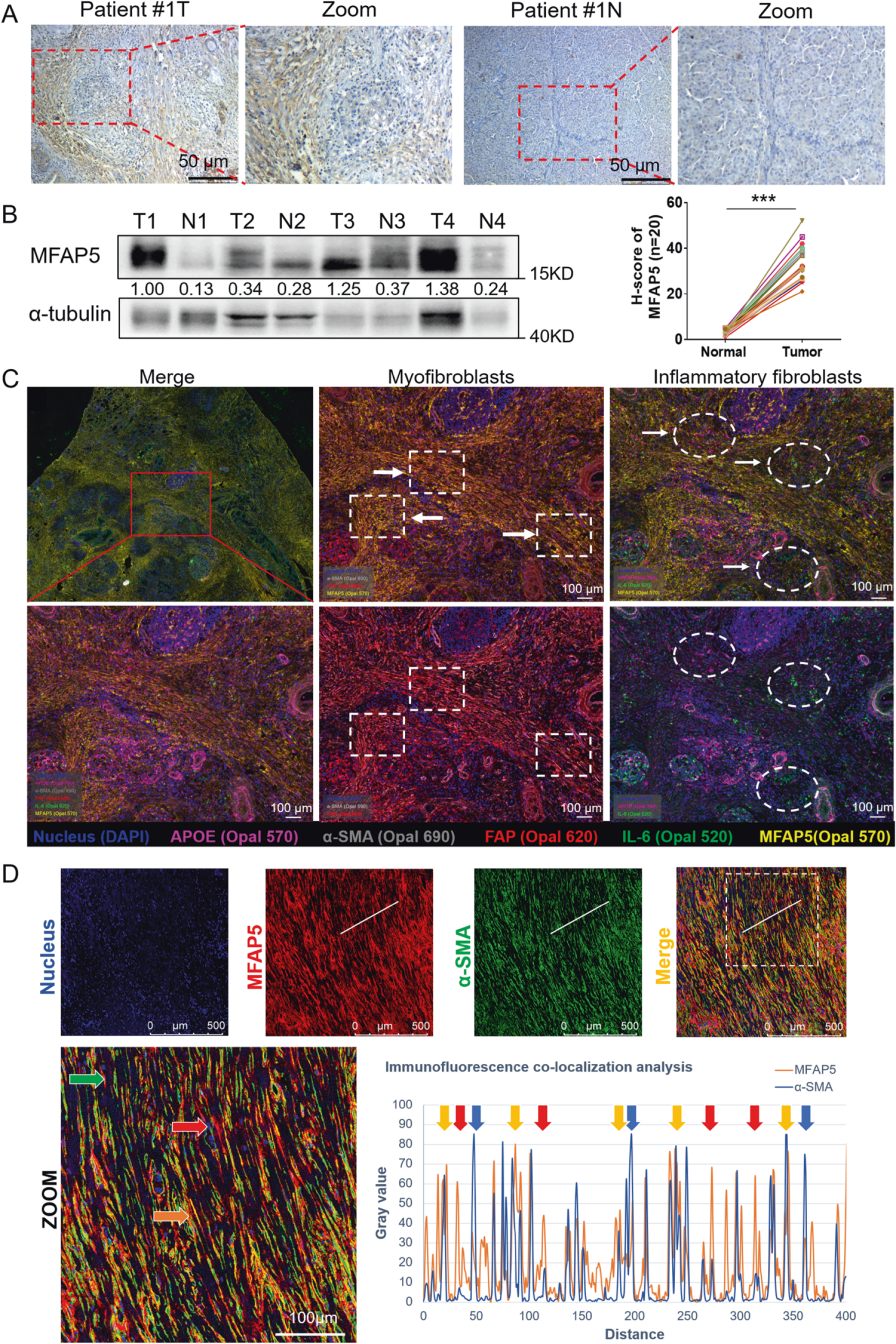
Expression features and prognostic signatures of MFAP5 in PDAC. **A** Representative images of MFAP5 expression in paraffin embedding pancreatic tumor (T) and matched adjacent pancreatic tissues (N) with IHC staining (*n* = 20) (*P* < 0.001). The paired scatter plot of H-score concerning MFAP5 staining was shown. Additional IHC pictures are shown in Fig. S1B. Scale bar: 50 μm. **B** Western blotting analysis of MFAP5 protein in paired PDAC tumor (T) and para-cancerous tissues (N). Gray values of the protein bands were calculated with Image J and the relative expression level were labeled. **C** Representative images of multiplex immunofluorescence staining on PDAC tissue. The channels are nucleus (DAPI, blue), APOE (Opal 780, fuchsine), α-SMA (Opal 690, gray), FAP (Opal 620, red), IL-6 (Opal 520, green) and MFAP5 (Opal 570, yellow). The rectangular frame indicates myofibroblasts enriched with α-SMA and FAP whereas the oval frame indicates inflammatory fibroblasts overexpressing APOE and IL-6. Comparison of two pictures in the same column shows the regional localization and expression of MFAP5. Scale bar: 100 μm. **D** Double immunofluorescence staining of nuclear (blue), MFAP5 (red) and α-SMA (green). The dashed box indicates the enlarged area and arrows indicate representative staining cells. Scale bar: 100 μm. White lines indicated area of the immunofluorescence co-localization analysis. In the curve graph, arrows indicate higher expression of MFAP5 (red), higher expression of α-SMA (blue) and co-expression of both proteins (yellow). The data were analyzed by a two‐tailed unpaired Student’s *t*‐test (**A**). ***P < 0.001.

In TCGA database, elevated mRNA expression of overall MFAP5 in tumor tissues indicated both poorer overall survival (OS) and relapse-free survival (RFS) of PDAC patients (Fig. S1A). We used surgically excised samples (*n* = 20) for IHC detection of MFAP5, and significantly higher expression level of MFAP5 was shown in cancerous tissues, especially the stromal regions, than in the matched adjacent pancreatic tissues (Figs. 1A and S1B, *P* < 0.001). Additionally, overexpression of MFAP5 protein was also observed in PDAC tumor tissue compared with paired para-cancerous tissues in western blotting (Fig. 1B). To assess the correlation between MFAP5 expression level and clinical outcomes of PDAC patients, corresponding clinicopathological information of the surgically excised tumor tissues on the array were collected, and data showed that high-level of MFAP5 was associated with nerve invasion, higher tumor TNM stages and poorer differentiation (Fig. S2A; Table S1). Apart from these findings, clinicopathological analysis of the in-house serum samples showed that high-level serous MFAP5 was associated with higher CEA level and poorer tumor differentiation of PDAC patients (Table S2). Thus, overall expression of MFAP5 in tumor tissues was correlated with a poor prognosis in patients with PDAC.

To assess MFAP5 expression in different cell types, normal pancreatic cell line HPNE, CAFs (including activated a-PSC, CAFs and resting q-PSC induced by all trans retinoic acid (ATRA)) and tumor lines were collected and screened. MFAP5 was highly expressed in CAF lines in comparison to the tumor or normal ones (Fig. S1C), which was consistent with the single-cell sequencing data of pancreatic tissues where MFAP5 was mainly expressed in fibroblasts (https://www.proteinatlas.org) (Fig. S2B). Furthermore, multiplex immunofluorescence staining was applied to evaluate the differential expression of MFAP5 in myofibroblasts and inflammatory fibroblasts in human PDAC tissues. As shown in Fig. 1C, MFAP5 was mainly enriched in myofibroblasts (*α*-SMA^high^ FAP^high^) and was relatively decreased in inflammatory fibroblasts (APOE^high^ IL-6^high)^, which indicated MFAP5 was likely to overexpress in myofibroblasts. In addition, in the double immunofluorescence staining of MFAP5 (red) and α-SMA (green) in human PDAC tissues, co-expression of MFAP5 and *α*-SMA was shown in most CAFs (Fig. 1D). Even in certain CAFs with relatively low fluorescence intensity of *α*-SMA, MFAP5 staining still prevailed. These results suggested the roles of MFAP5 in indicating CAFs, especially myofibroblasts in a faintly active or relatively quiescent state. Besides, MFAP5 abundance was significantly related to expression of collagen-synthesis-related genes (Fig. S2C). Collectively, MFAP5 was mainly enriched in myofibroblasts and was likely to involve in CAFs activation.

### MFAP5 knockdown attenuates CAFs activation and restrains tumor progression

**Fig. 2:**
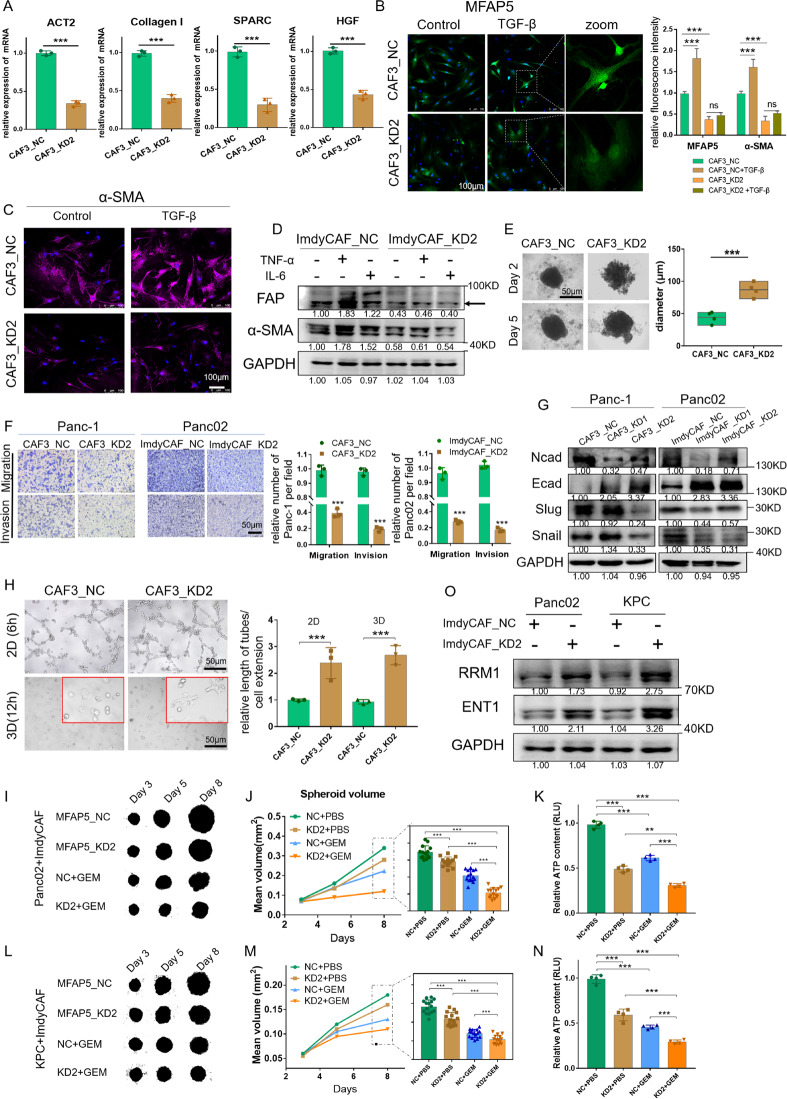
MFAP5 knockdown inhibits CAFs activation and tumor progression in vitro. **A** Relative expression of CAF activation-related genes detected with qRT‐PCR. **B**, **C** Immunofluorescence staining of MFAP5 and α-SMA in human MFAP5_KD or control CAFs with or without TGF-β (5 ng/ml for 24 h). Scale bar: 100 μm (**D**) Western blotting assay of CAF activation in MFAP5_KD or control ImdyCAFs treated with TNF-α or IL-6 (10 ng/ml for 48 h). Arrow indicates the true protein band. **E** Morphological characteristics and changes in diameter measured in day 2 and day 5 in spheroid formation assay. Statistical of diameter at day 2 is shown on the right. Scale bar: 50 μm. **F** Trans-well experiment indicates migration and invasive capabilities of Panc-1 and Panc02 in the conditioned medium. Panc-1 was co-cultured with CAF3 control or KD lines, and Panc02 was cultured with ImdyCAF control or KD lines. Scale bar: 50 μm. **G** Western blot assay of EMT-related proteins in Panc-1 and Panc02 from the cocultured systems. **H** Representative images of 2D/3D tube formation assays of HUVEC cells in cocultured system and statistical results. Scale bar: 50 μm. **I**–**N** Representative images of heterospheroids derived from Panc02 or KPC and stable transfected ImdyCAFs after treatment of gemcitabine (10 μM) (**I** and **L**), and the statistical results of spheroid volume (**J** and **M**) and cell viability (**K** and **N**). **O** Western blotting assay of gemcitabine transport proteins in Pand02 and KPC after cocultured with conditioned medium for 48 h. Gray values of the protein bands were calculated with Image J and the relative expression level were labeled. The data were analyzed by a two‐tailed unpaired Student’s *t*‐test (**A**, **B**, **E**, **F**, **G**, **H**, **J**, **K**, **M** and **N**). Error bars, means ± SD of three independent experiments. *P < 0.05, **P < 0.01, ***P < 0.001, ns not significant.

To further identify roles of MFAP5 in CAFs, we extracted and cultured human primary CAFs (CAF3) derived from the PDAC patient, and mouse primary CAF cell line (ImdyCAF) derived from spontaneous pancreatic tumors in transgenic mouse (Kras^LSL-G12D^, Trp53^LSL-R172H^, Pdx1-Cre (KPC) mice) (Fig. S3A). Then stable genetic knockdown (KD) of MFAP5 via lentivirus infection (CAF3_KD/ImdyCAF_KD) was performed and validated. Interestingly, decreased expression of α-SMA and activation markers of CAFs was observed in MFAP5 deficient CAFs (Figs. 2A, S3B, C). When stimulated by TGF-*β*, TNF-*α* or IL-6, MFAP5_KD CAFs showed lower expression of α-SMA compared with control groups (Fig. 2B–D). Meanwhile, compared with normal CAFs, MFAP5 deficiency resulted in larger diameter with less compact structure of 3D spheroids formation after 5 days of culturing (Fig. 2E). These data highlighted the function of MFAP5 concerning CAF activation and stroma formation. Additionally, knockdown of MFAP5 also impaired proliferation and metastasis abilities of CAFs/ImdyCAFs (Fig. S3D–I). PDAC cells were surrounded by abundant CAFs and matrix in vivo [12]. Co-culture systems of CAFs and tumor cells were built. Both Panc-1 and Panc02 cell lines showed reduced growth rate, retarded migratory and invasive capabilities when co-cultured with MFAP5_KD CAFs in comparison to normal CAFs in vitro (Figs. 2F, S4A–D). Changes in epithelial-mesenchymal transition (EMT)-related proteins included reduced N-cad, Slug, Snail and elevated E-cad indicated increasing mesenchymal like phenotype and impaired migration abilities (Fig. 2G). Therefore, MFAP5 was involved in CAF activation, tumor proliferation and metastasis in vitro.

As shown in Fig. 2H, tube formation ability of HUVEC were enhanced when they were cocultured with MFAP5_KD CAFs in both 2D and 3D coculture systems (Fig. S4E). Since increased intratumor vessels could improve drug delivery, we investigated whether knockdown of MFAP5 in CAFs could enhance chemotherapeutic effects. In vitro, the 3D heterospheroids systems containing tumor cells and stably transfected ImdyCAFs were built, and the average size of the spheroids containing MFAP5_KD ImdyCAFs cotreated with gemcitabine was significantly smaller than the other subgroups in both KPC and Panc02 cell lines, which was consistent with changes of spheroids’ growth viabilities using adenosine triphosphate (ATP) analysis (Fig. 2I–N). Notably, gemcitabine transport protein RRM1 and ENT1 on tumor cells, which could induce more absorption of gemcitabine into tumor cells to exert chemical killing effect [27, 28], were upregulated when cocultured with MFAP5_KD CAFs (Fig. 2O). Collectively, MFAP5 knockdown could promote tube formation of HUVEC and intra-tumoral transport of gemcitabine to enhance the chemotherapeutic effect.

### MFAP5^high^CAFs contribute to the desmoplastic and immunosuppressive TME in vivo

**Fig. 3:**
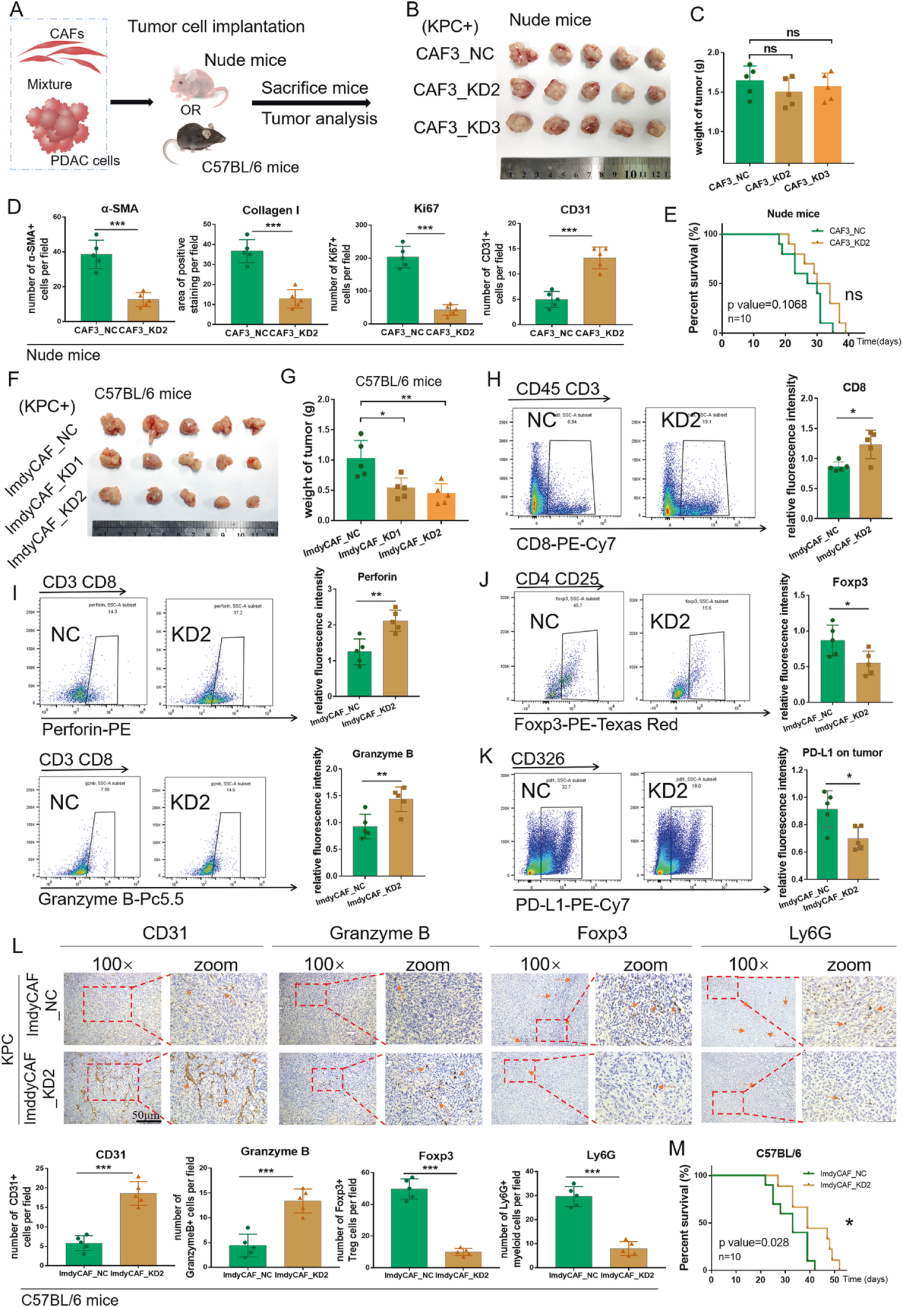
Inhibiting MFAP5^high^CAFs remodels the immunosuppressive microenvironment in vivo. **A** Schematic procedures of xenograft models built with stable transfected CAFs and PDAC cells co-injected into the pancreas of either nude or C57BL/6 mice. **B**, **C** Images and statistical results of tumor weight of isolated tumors derived fromKPC+CAFs after mice were sacrificed at day 28. A total of 15 mice were analyzed (5 mice for each group individually). **D** Histograms showing markers indicating CAF activation (α-SMA and Collagen I), tumor proliferation (Ki67), angiogenesis (CD31) in IHC staining in Fig. S4D. **E** Survival curves of mice in nude mice (*p* = 0.1068) bearing KPC xenograft tumors. A total of 20 mice were included (10 mice for each group individually). **F**, **G** Images and statistical results of tumor weight of isolated tumors derived from KPC + ImdyCAFs after mice were sacrificed at day 28. **H**–**K** Flow cytometry analysis of CD8 (PE-Cy7), Perforin (PE), Granzyme B (Pc5.5), Foxp3 (Texas Red) and tumoral PD-L1 (PE-Cy7) in tumors derived from C57BL/6 mice. The top arrow indicates the upper gate logic. A total of 10 mice were analyzed (5 mice for each group individually). **L** Representative IHC staining images and statistical analysis (below) of functional infiltrating CD8 + T cells (Granzyme B+) and immunosuppressive cells (Foxp3+Tregs and Ly6G+ myeloid cells). Arrows indicate representative staining. Scale bar: 50 μm (M) Survival curves of mice in C57BL/6 mice (*p* = 0.1068) bearing KPC xenograft tumors. A total of 20 mice were included (10 mice for each group individually). The data were analyzed by a two‐tailed unpaired Student’s *t*‐test (**C**, **D**, **G**, **H**, **L**). Error bars, means ± SD of three independent experiments. *P < 0.05, **P < 0.01, ***P < 0.001, ns not significant.

To examine roles of MFAP5 in stromal reconstruction in vivo, a mixture of tumor cells and homogenous MFAP5_KD or control CAFs were orthotopically co-transplanted to nude mice or C57BL/6 mice (Fig. 3A). The tumor-promoting effects of CAFs were pre-validated in C57BL/6 mice (Fig. S5A). Interestingly, in nude mice models, tumors originated from MFAP5_KD CAFs group were moderately decreased compared with that from control CAFs group (Figs. 3B–E, S5B–D), whereas the difference was more significant in C57BL/6 mice with normal immune function (Fig. 3F, G) (nearly 40% more reduction in tumor weight with similar gene knockdown efficiency in mRNA and protein levels in both CAF cell lines). The differences in tumor weight in nude mice and C57BL/6 mice indicated involvement of the immune system (Fig. 3C, G). Additionally, decreased expression of *α*-SMA, Collagen I, Ki67 and elevated expression of CD31 were observed in MFAP5 deficient tumors (Figs. 3D, S5D), which indicated that MFAP5 deficiency could reshape the compact stroma by inhibiting CAF activation and promoting angiogenesis. Moreover, in order to distinguish the implanted ImdyCAFs from the local stroma, ImdyCAFs stably transfected with control vectors with RFP tag were co-injected in to C57BL/6 mice, and the generated tumors were analyzed with immunofluorescence staining (Fig. S5E).

In terms of immune regulation, flow cytometry and IHC analysis revealed that MFAP5 deficiency increased infiltration of Granzyme B^+^Perforin^+^CD8^+^T cells, reduced distribution of immunosuppressive cells (Foxp3^+^Tregs and Ly6G^+^ myeloid cells) (Fig. 3H–L), and decreased tumoral PD-L1 expression (Fig. 3K) by flow cytometry and immunohistochemical analysis. Moreover, in comparison with nude mice, MFAP5 knockdown in C57BL/6 mouse models showed improved survival outcomes, with median survival 28.5 vs 32 days for nude mice in Fig. 3E (not significant), and 33 vs 39 for C57BL/6 mice in Fig. 3M (*P* = 0.028).

### MFAP5 promotes HA synthesis via RCN2/ERK/STAT1 axis

**Fig. 4:**
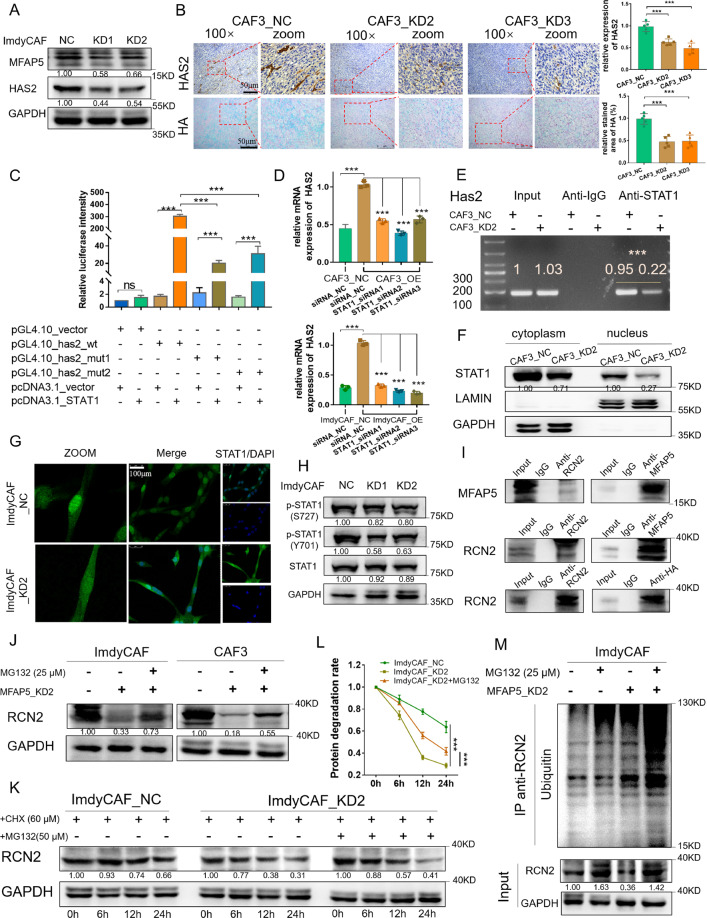
MFAP5 promotes synthesis of HA via maintaining stability of RCN2. **A** HAS2 expression in MFAP5_KD or control CAFs via western blot assay. **B** Representative images of IHC staining of HAS2 and chemical staining of HA in paraffin embedded tumor sections derived from mice models. Scale bars, 50 μm. **C** Relative fluorescence intensity in 293 T cells co-transfected with constructed luciferase reporter plasmids. **D** Relative expression of HAS2 when STAT1 was interfered in MFAP5_OE or control CAFs with qRT-PCR. **E** Representative image of HAS2 fragments in agarose gel in the ChIP assay. **F**, **G** Spatial distribution of STAT1 in MFAP5_KD or control CAFs via nucleus-cytoplasm separation assay (**F**) and immunofluorescence staining (**G**). Scale bars, 100 μm (**H**) Western blot analysis of phosphorylated STAT1 (S727 and Y701) in MFAP5_KD or control CAFs. **I** Western blot analysis of protein combination following immunoprecipitation in ImdyCAFs. **J** Western blot analysis of RCN2 expression in MFAP5_KD or control CAFs treated with MG132 (25 μM, 24 h). **K** Western blot of protein stability and statistical results (**L**) of CAFs treated with MG132 (50 μM, 24 h) and/or cycloheximide (CHX, 25 μM, 24 h). **M** Ubiquitination combination assay of RCN2 in MFAP5_KD or control CAFs treated with MG132 (25 μM, 24 h). Gray values of the protein bands were calculated with Image J and the relative expression level were labeled. The data were analyzed by a two‐tailed unpaired Student’s *t*‐test (**A**, **D**, **E**, **G**, **H**, **M**). Error bars, means ± SD of three independent experiments. *P < 0.05, **P < 0.01, ***P < 0.001, ns not significant.

To demonstrate the underlying mechanism of crosstalk between CAFs and PDAC cells, RNA-seq analysis was performed. Notably, hyaluronate synthase-2 (*HAS2*) was significantly downregulated in transcriptional level in MFAP5_KD groups (Fig. S6A, B). Changes in various cancer-related pathways were also highlighted (Fig. S6C). Downregulation of HAS2 was verified via western blot and qRT-PCR assay (Figs. 4A, S6D). Bioinformatics analysis indicated the positive correlation between MFAP5 and HAS2 (R = 0.57) (Fig. S6E). In addition, both MFAP5 and HAS2 were overexpressed in PDAC tumors compared with adjacent normal tissues, and MFAP5 deficiency in CAFs led to decreased HAS2 expression as well as reduced HA deposition in vivo (Fig. 4B). In JASPAR database, STAT family members were among the candidates that could directly bind to promoter regions of HAS2, and gene co-expression correlation between STAT1 and HAS2 was the most significant (R = 0.41) (Fig. S6F). These findings were further validated with double luciferase reporter gene assays (Figs. 4C, S6G, H). Additionally, upregulated *HAS2* due to overexpressed MFAP5 was partially restored when STAT1 was interfered in CAFs (Fig. 4D). Besides, reduced binding of STAT1 on promoter region of *HAS2* was also observed in MFAP5_KD CAFs compared to control CAFs via ChIP assay (Fig. 4E). These data indicated roles of MFAP5 in regulating binding of STAT1 and HAS2. Moreover, nuclear translocation of STAT1 was significantly reduced in MFAP5_KD CAFs compared with normal CAFs (Fig. 4F, G). Collectively, MFAP5 modulated expression of HAS2 via nuclear translocation of STAT1 in CAFs.

Phosphorylation of STAT1 promotes its translocation from cytoplasm to nucleus (mainly phosphorylated at site Y701 or S727) [29, 30]. Decreased phosphorylated STAT1 (more significant at Y701 site) was observed in MFAP5-KD CAFs (Fig. 4H). To explore the underlying mechanism, proteins binding with MFAP5 in CAFs were investigated through LC-MS/MS (Fig. S6J). Among the candidates, the Ca^2+^-binding protein Reticulocalbin 2 (RCN2) has been reported as a direct activator of MAPK/ERK signaling pathways, which could further induce phosphorylation of STAT families [31, 32]. To support this speculation, biological binding of MFAP5 and RCN2 in CAFs was validated by co-immunoprecipitation (co-IP) assays (Figs. 4I, S6K). The fact that MFAP5 knockdown mainly reduced RCN2 in protein levels instead of mRNA levels indicated that MFAP5 might involve in posttranslational modification of RCN2 protein. As shown in Fig. 4J–L, decreased RNC2 and shorter half-life of protein degradation caused by MFAP5 deficiency were both partially rescued by proteasome inhibitor, concomitant with consequent changes in ERK pathways (Fig. S6L). In addition, activation roles of RCN2 on ERK pathways were further verified in Fig. S6M. These results indicated that MFAP5 prevents ubiquitination-mediated proteasomal degradation of RCN2. Moreover, increased combination of ubiquitination on RCN2 was also observed in MFAP5-KD CAFs compared with normal CAFs (Fig. 4M). And application of MG132 could reverse the decreased nucleus STAT1 due to MFAP5 deficiency (Fig. S6N). Collectively, MFAP5 deficiency inhibits aberrant activation of ERK pathways via proteasome-mediated protein degradation of RCN2 and further decrease synthesis of HA via STAT1.

### MFAP5 elicits tumoral PD-L1 expression via CXCL10

**Fig. 5:**
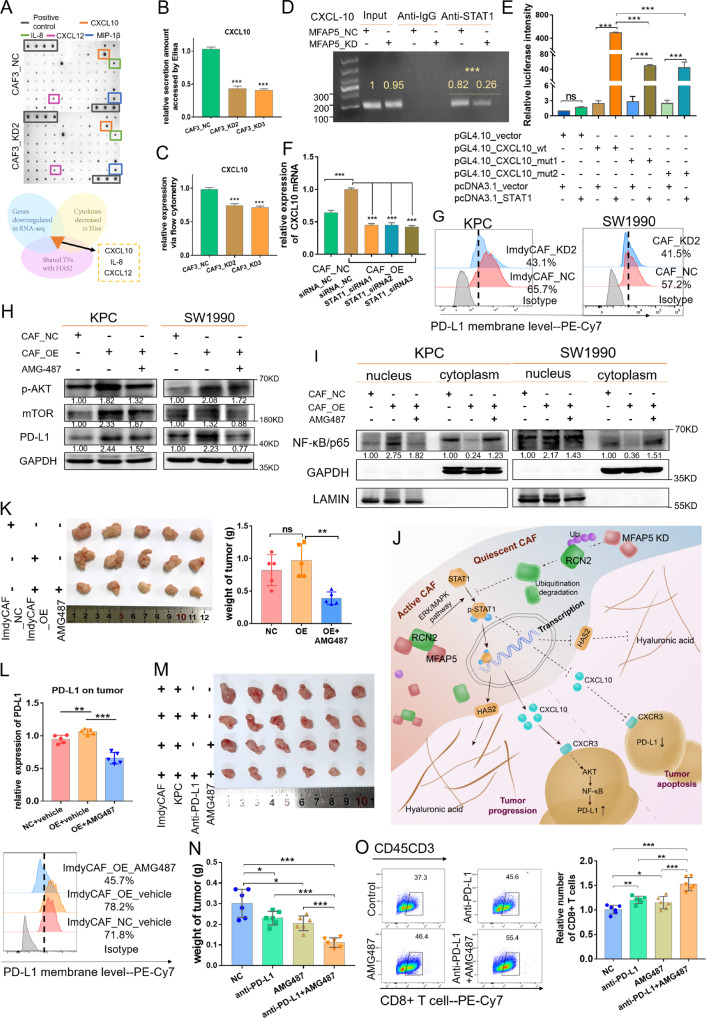
Blockade of CXCL10 restored the upregulation of tumoral PD-L1 resulted from MFAP5 overexpression in CAF. **A** Cytokine array and venn diagram shows screening process of CXCL10. Representative differential dots were framed. **B**, **C** Validation of changes in CXCL10 via Elisa assay (**B**) and flow cytometry (**C**). **D** Representative image of CXCL10 fragments in the agarose gel of the ChIP assay. **E** Relative fluorescence intensity in 293 T cells co-transfected with constructed luciferase reporter plasmids. **F** Relative expression of CXCL10 when STAT1 is interfered in MFAP5_OE or control CAFs by qRT-PCR. **G** Flow cytometry analysis of PD-L1 (PE-Cy7) on KPC and SW1990 cell lines cocultured MFAP5_KD or control CAFs (48 h) in vitro. **H** Western blot analysis of mTOR, p-AKT and PD-L1 in PDAC cell lines in cocultured systems with or without AMG487 (5 nM, 48 h). SW1990 was co-cultured with CAF3 control or OE lines, and KPC was cultured with ImdyCAF control or OE lines. **I** Spatial distribution of NF-κB/p65 in tumor cells cultured in conditioned CAF medium with or without AMG487 (5 nM, 48 h) by western blotting. SW1990 was co-cultured with CAF3 control or OE lines, and KPC was cultured with ImdyCAF control or OE lines. **J** Sketch Figure indicating underlying molecular mechanisms of MFAP5 in regulating HAS2 and CXCL10 as well as PD-L1 in tumor cells. **K** Images and statistical tumor weight of isolated tumors derived from KPC and MFAPF_OE or control ImdyCAFs with administration of AMG487 (5 mg/kg). A total of 15 mice were analyzed (5 mice for each group individually). **L** Flow cytometry analysis of PD-L1 (PE-Cy7) on tumor cells of isolated tumors in (**J**). **M**, **N** Images and statistical analysis (**N**) of isolated tumors. C57BL/6 mice were orthotopically co-injected with ImdyCAFs and KPC cells combined with administration of AMG487 (5 mg/kg) and anti-PD-L1 antibody (200 μg/mouse) every three days. **O** Flow cytometry analysis of CD8 (PE-Cy7) on tumor cells of isolated tumors in (**M**). Gray values of the protein bands were calculated with Image J and the relative expression level were labeled. The data were analyzed by a two‐tailed unpaired Student’s *t*‐test (**B**, **C**, **D**, **E**, **F**, **K**, **N**, **O**). Error bars, means ± SD of three independent experiments. *P < 0.05, **P < 0.01, ***P < 0.001, ns not significant.

We noticed that certain cytokines and chemokines were downregulated in RNA-seq analysis when MFAP5 was knockdown in CAFs. Cytokine array was further performed to screen and identify cytokines as possible immune regulating messengers between CAFs and tumor cells. The intersecting candidates of the RNA-seq and Cytokine array was further verified by in vitro experiments. In Fig. 5A, a significant reduction of CXCL10 was observed in MFAP5_KD CAFs compared with control CAFs, which was further validated with flow cytometry and Elisa assay in vitro (Fig. 5B, C), whereas reduction of other cytokines including IL-8 and CXCL12 was failed to be detected (Fig. S7A). Previous studies have reported the association between STAT1 and CXCL10 in PDAC and colorectal cancer [33, 34]. We confirmed the binding sites of STAT1 on promoter regions of *CXCL10* in PDAC CAFs via double fluorescein reporter gene assay and ChIP assay (Figs. 5D, E, S7B). MFAP5 deficiency led to decreased binding of STAT1 on *CXCL10* promoter region in comparison with control CAFs. The upregulated CXCL10 caused by overexpressed MFAP5 was restored when STAT1 was interfered (Fig. 5F). These data indicated MFAP5 also regulates expression of CXCL10 via STAT1.

CXCL10 is a potent angiogenesis inhibitor [35]. In our present study, antagonist AMG 487 was applied to interfere the binding between CXCL10 and CXCR3. Notably, promoted cell cycle and changes in EMT-related proteins of Panc-1 and Panc02, as well as the impaired tube formation ability in HUVEC attributed to MFAP5_OE CAFs was partially reverted when CXCL10 was blocked (Fig. S7C–E). CXCL10 was previously reported to promote tumoral PD-L1 expression in cervical cancer and gastric cancer [36, 37]. Given that MFAP5 inhibition downregulated PD-L1 expression on tumor cells in vivo (Fig. 3K), we further demonstrated downregulation of PD-L1 on tumor cells when cocultured with MFAP5_KD CAFs in vitro (Fig. 5G). Moreover, changes in downstream AKT/mTOR signaling pathways, nuclear translocation of NF-κB and total PD-L1 protein level could also be partially rescued by AMG487 (Fig. 5H, I). These results indicated that MFAP5 in CAFs might regulate tumoral PD-L1 expression via CXCL10/CXCR3/AKT/NF-κB axis (Fig. 5J).

Furthermore, in xenograft models, the enhanced tumor proliferation (Ki67) and decreased angiogenesis (CD31) due to MFAP5 overexpression in CAFs was also partially reverted by application of AMG487, along with increased Granzyme B^+^CD8^+^T cells infiltration (Figs. 5K, S7F–H). Notably, upregulated PD-L1 on tumor cells caused by MFAP5_OE CAFs was also partially reversed when CXCL10 was blocked in vivo (Fig. 5L). In addition, we further carried out in vivo experiments with KPC tumor cells alone (without ImdyCAFs) and treated the xenograft mice with AMG487 every three days. As shown in Fig. S7I, approximately 40% reduction in tumor weight was observed in the AMG487 treated group compared with the control group, which showed no obvious extra benefit compared with results in models injected with CAFs (Figs. 5K, S7G). Besides, mice with AMG487 treatment also showed survival benefit compared with the control group (Fig. S7I, *P* = 0.0275). Since CXCL10 could influence expression of tumoral PD-L1, the combined administration of AMG487 and anti-PD-L1 antibody was further performed in xenograft models (Fig. S8A). Interestingly, the combined therapy could inhibit tumor growth and increase infiltrated CD8^+^T cells compared with any of the mono-drug subgroups (Fig. 5M–O). In addition, body weight and blood biochemistry indicators of mice were detected and no obvious drug toxicity was observed (Fig. S8B, C). These data highlight that AMG487 could sensitize PDAC tumors to PD-L1-based immunotherapy.

### Targeting MFAP5^high^CAFs synergizes with immunochemotherapy

**Fig. 6:**
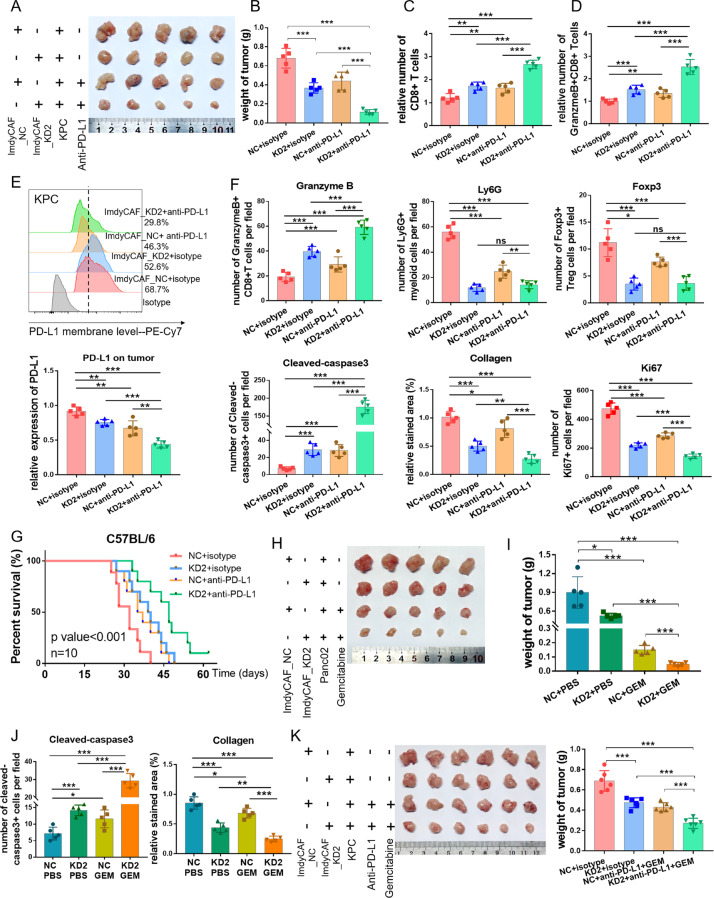
MFAP5 inhibition in CAFs synergizes with PD-L1-based immunotherapy. **A**, **B** Images and statistical tumor weight of isolated tumors derived from mice co-injected with KPC and MFAP5_KD or control CAFs and administrated with anti-PD-L1 antibody. A total of 20 mice were included (5 mice for each group individually). **C**–**E** Flow cytometry analysis of CD8 + T cells, Granzyme+CD8 + T cells and tumoral PD-L1 level of tumors in (**A**). **F** Statistical analysis of Granzyme B, CD31, Foxp3, Cleaved-caspase3 and Ki67 via IHC staining, and collagen detection by picrosirius red chemical staining of tumors in (**A**). **G** Survival curves of immunocompetent C57BL/6 with KPC tumor bearing co-intervention of MFAP5 inhibition and/or anti-PD-L1 antibody (*p*-value < 0.001). A total of 40 mice were analyzed (10 mice for each group individually). **H**, **I** Images and statistical tumor weight of isolated co-injected Panc02 tumors after mice were sacrificed at day 28. A total of 20 mice were analyzed (5 mice for each group individually). **J** Statistical analysis of Cleaved-caspase3 by IHC staining and collagen deposition by chemical staining (picrosirius red) in tumors in (**H**). **K** Images and statistical tumor weight of isolated tumors derived from mice co-injected with KPC and MFAP5_KD or control CAFs and administrated with anti-PD-L1 antibody as well as gemcitabine every three days. A total of 24 mice were included (6 mice for each group individually). The data were analyzed by a two‐tailed unpaired Student’s *t*‐test (**B**, **C**, **D**, **E**, **F**, **I**, **J**, **K**). Error bars, means ± SD of three independent experiments. *P < 0.05, **P < 0.01, ***P < 0.001, ns not significant.

Furthermore, combined effect of MFAP5^high^CAFs inhibition and ICB therapy was also demonstrated in vivo. A mixture of KPC and MFAP5_KD or control CAFs was orthotopically co-injected in C57BL/6 mice, and both the anti-PD-L1 and isotype antibody were regularly administrated (Fig. S9A). Notably, MFAP5^high^CAFs inhibition in combination with anti-PD-L1 treatment significantly suppressed tumor proliferation with a reduction in tumor weight by over 70%, along with reduced collagen deposition and immunosuppressive cells including Foxp3^+^Treg cells and Ly6G^+^myeloid cells, increased infiltration of cytotoxicity Granzyme B^+^CD8^+^T cells and obvious tumor cell apoptosis (Cleaved-Caspase3), in comparison to any of the mono-treated subgroups (Figs. 6A–F, S9D–E). To further confirm involvement of CD8^+^T cells in MFAP5_KD subgroups, CD8 deletion assay was performed in vivo and the tumor volume in MFAP5 deficient subgroup was restored when CD8 + T cells were neutralized with anti-CD8 antibody (Figs. S9F, G). Besides, similar phenomena were observed and validated in Panc02-bearing xenograft mice (Fig. S9B, C). Moreover, the combined treatment led to improved outcomes with prolonged survival duration compared with any of the mono-treatment groups, with median survival 32 vs 39.5 vs 36 vs 47 days for ImdyCAF_NC, KD2, NC + PD-L1 and KD2 + PD-L1 subgroups, respectively (*P* < 0.001) (Fig. 6G).

**Fig. 7:**
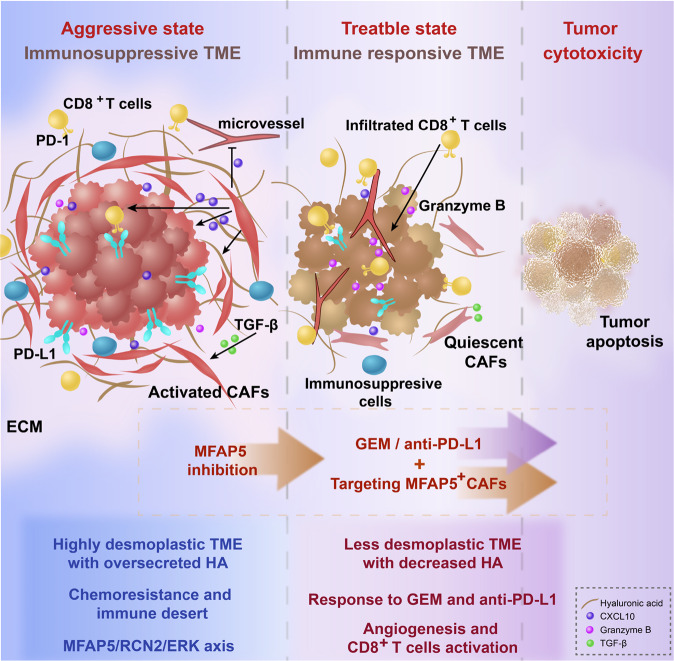
Sketch Figure indicating roles of MFAP5 in remodeling the desmoplastic and immunosuppressive TME of PDAC.

In addition, xenograft models in vivo further estimated the chemotherapy enhancing effect of inhibiting MFAP5^high^CAFs (Fig. S10A). Obviously, MFAP5 knockdown could enhance the therapeutic effect of gemcitabine in both subgroup tumors injected with KPC or Panc02 (Figs. 6H, I, S10B, C). In addition, enhanced pro-apoptosis and antifibrotic effect were observed in the combined treatment group (Figs. 6J, S10D). Collectively, inhibiting MFAP5^high^CAFs could enhance chemotherapeutic effect of gemcitabine. Furthermore, triple combination of gemcitabine, anti-PD-L1 antibody and MFAP5 inhibition was performed in xenograft mice models to mimic the clinical regimens. Notably, MFAP5 knockdown was in synergy with PD-L1 based immunotherapy and gemcitabine treatment to inhibit tumor growth potently (Fig. 6K). In conclusion, targeting MFAP5^high^CAFs sensitizes pancreatic tumor to PD-L1-based immunochemotherapy via remodeling the desmoplastic and immunosuppressive TME (Fig. 7).

## Discussion

The desmoplastic and immunosuppressive TME in PDAC is vicious for containing large amounts of CAFs and stromal components including collagens, HA, fibronectin [38, 39]. Over the past few years, CAF heterogeneity has been explored, and single cell analysis has shed light on the complex characteristics and functions of CAFs including myCAFs, inflammatory CAFs and antigen-presenting CAFs, etc [40, 41]. Considering that the available single cell data of PDAC tumors can only attribute MFAP5 to the fibroblast subgroup, rather than further precisely classify it into myofibroblasts or inflammatory CAFs. Our multiplex immunofluorescence results reveal that MFAP5 is mainly overexpressed in myofibroblasts, which are one of the major sources of HA. However, failure of the phase III clinical trial targeting HA reminds us that it is necessary to maintain the normal structure of the stroma while eliminating the carcinogenic components. Notably, in the 3D spheroids culturing in vitro, MFAP5 deficiency led to decreased secretion of collagens and HA by CAFs and larger diameter with less compact structure of the spheroids. As reported, the majority of interstitial PDAC stromal fluid was present in a gel-fluid phase, and the intra-tumoral pressure was mainly composed by interstitial fluid pressure (mainly from HA) and solid stress (mainly from cancer cells proliferation) to maintain a balance [42–44]. Complete elimination of HA is not recommended as this might result in disruption of the pressure balance and matrix structure. MFAP5 deficiency in CAFs could remodel the matrix via reducing HA secretion and inhibiting tumor cell proliferation simultaneously, in avoidance of disruption of matrix balance when merely eliminating HA. Additionally, compared with the acknowledged CAF activation indicator α-SMA, MFAP5 was also expressed in CAFs with relatively low expression of α-SMA. Therefore, we speculate that detection of MFAP5 replenishing α-SMA may increase the sensitivity and specificity in CAF identification and classification, especially for CAFs in quiescent or faintly active state.

CAFs are inherently resistant to chemotherapies [45]. Thus, the compact stromal shell shaped by CAFs and their secretions can protect tumor cells from chemotherapeutics, such as gemcitabine. In our results, inhibition of MFAP5 in CAFs decreases HA secretion and elicits angiogenesis, breaking the matrix shell (physical barrier) and making PDAC accessible to chemotherapeutics. Mechanistically, MFAP5 inhibition reduces expression and secretion of potent angiogenesis inhibitor CXCL10. Increased angiogenesis promotes drug delivery to tumor niches. Furthermore, upregulation of Gemcitabine transport protein (ENT1 and RRM1) on tumor cells leads to increased drug bioavailability of PDAC cells. In a word, the non-immune functions of MFAP5 reshape the dense stroma to a relatively loose state, providing a structural basis for chemotherapeutics penetration.

Previously, Yeung et al. have reported the non-immune roles of MFAP5 concerning fibrosis in ovarian cancer and pancreatic cancer [23]. It is also acknowledged that PDAC tumor cells are quarantined from cytotoxic CD8^+^T cells by dense stroma [7]. Therefore, we try to investigate the immunoregulation effect of MFAP5. Surprisingly, we observed more cytotoxic CD8^+^T cells infiltrated into the center area under MFAP5 deficiency, and synergistic effect of MFAP5^high^CAFs inhibition and PD-L1-based immunotherapy is testified in vivo. Studies have reported tumoral PD-L1 expression renders immune escape and CD8^+^T cells exhaustion [6, 46, 47]. Although PDAC is considered to be a less immunogenic cancer type with relatively low PD-L1 expression and CD8^+^T cells infiltration, PDAC cases characterized by high levels of TILs have been reported to be associated with prolonged overall survival [48]. The PD-L1^-^/CD8^high^ PDAC subtype indicates a more favorable prognosis [49]. Thus, remodeling the dense stroma, downregulating tumoral PD-L1, and increasing TILs infiltration are of vital importance for PDAC patients to improve immune response and clinical prognosis.

In summary, we first uncover that MFAP5 inhibition in CAFs promotes immune response, and propose a unique clue of targeting MFAP5^high^CAFs in synergy with PD-L1-based immunochemotherapy in systematic treatment of PDAC patients in clinic. Nevertheless, further efforts are still warranted in exploring credible monoclonal antibodies or small molecular inhibitors targeting MFAP5 in CAFs.

## Materials and methods

### Patient specimens

Clinical human PDAC tumor tissue and adjacent normal tissues were surgically excised and collected from the Department of Hepatobiliary and Pancreatic. A total of 140 clinical PDAC tissue samples and 50 serum samples with complete clinicopathological information were included for further analysis. Both paired tissues and serum were collected and stored at −80 °C. In addition, corresponding clinicopathological information including sex, age, tumor grade, serum cancer antigen levels of CA19-9 and CA125), as well as clinical tumor-node-metastasis (TNM) stages were artificially collected, and patients with complete information were included in the following analysis. MFAP5 expression low or high subgroups were divided according to H-score of the IHC staining intensity or the relative amount of MFAP5 detected in serum via Elisa assay. Notably, the project was approved by the Institutional Review Board at First Affiliated Hospital, School of Medicine, Zhejiang University, and patients involved in the study all signed the informed consent.

### Isolation of primary CAFs and cell culture

Primary CAF3 and ImdyCAF cell lines were isolated from tumors obtained from clinical PDAC patients or the (Kras^LSL-G12D^, Trp53^LSL-R172H^, Pdx1-Cre) (KPC) mice. Center area tissues were minced and digested with collagenase (Thermo Fisher Scientific). The centrifugated cell pellets were filtered and seeded in tissue culture dishes with specific complete SteCM culture medium. Single cells were screened and sorted by fluorescence-activated cell sorting (CD31^−^CD45^−^CD202b^-^cd326^−^cd324^−^), and further identification of spindle-shaped morphology and CAF-specific markers (especially α-SMA^+^, FAP^+^ and CK19^−^) were performed. The KPC cell line was kindly donated by Prof. Raghu Kalluri, which was isolated and cultured from the genetically engineered mouse model (Kras^LSL-G12D^, Trp53^LSL-R172H^, Pdx1-Cre) (KPC) mice. The other cell lines were obtained from ATCC (American Type Culture Collection). As previously described [46], cells were cultured in medium with 10% fetal bovine serum (Thermo Fisher Scientific) and 1% penicillin-streptomycin at 37 °C in a humidified atmosphere containing 5% CO2. In addition, negative mycoplasma contamination was routinely evaluated with the PCR assay, and all cell lines were identified using short tandem repeat analysis.

### Animal care and in vivo models and flow cytometry analysis

Both nude mice and C57BL/6 mice at 6–8 weeks old were purchased and maintained in SPF environment. All animal experiments were performed according to guidelines approved by the Ethics Committee of the First Affiliated Hospital, School of Medicine, Zhejiang University. In our present study, mice were randomly divided into different subgroups in each independent experiment. A mixture of homogeneous PDAC cells and stable transfected CAFs at a ratio of 1:2 was orthotopically co-injected into the pancreas to mimic the PDAC microenvironment. The injection volume was 20 μl containing 10 μl medium mixed with 10 μl Matrigel for each mouse. Briefly, Panc-1 (8 × 10^5^ cells) and MFAP5_KD or control human CAFs (1.6 × 10^6^ cells) were co-injected into nude mice. Besides, KPC or Panc02 (5 × 10^5^ cells) were mixed with MFAP5_KD or control mouse ImdyCAFs (1 × 10^6^ cells) for further co-injection in C57BL/6 mice. In the gemcitabine synergistic study, gemcitabine (50 mg/kg) and vehicle were intraperitoneally injected every three days for a total of 6 times of injection. In the restore experiment blocking binding of CXCL10 and CXCR3, AMG487 (5 mg/kg) and the vehicle were intraperitoneally given 3 times a week for two weeks. And in immunotherapy combination experiment, anti-mouse PD-L1 InVivoMAb (200 μg/mouse, Bio X Cell) and equal amount of IgG isotype control were intraperitoneally administrated every three days for a total of 6 times. Anti-mouse CD8 monoclonal mAbs (200 μg/mouse, Bio X Cell, InVivoPlus grade, clone 53–6.7) and equal amount of IgG isotype control were intraperitoneally administrated three times before tumor inoculation and every three days. In the final of each experiment, mice were sacrificed and tumors were harvested for following analysis. For survival analysis, mice models were established as described above with or without the PD-L1 treatment. Death event occurred in each group was recorded to plot the survival curves.

The harvested tumors were processed for following staining and flow cytometry analysis. Tumor tissues were dissociated and digested as single cells for flow cytometry analysis as previously described [46]. In our study, one part of cells was stained with CD326 to assess expression of PD-L1, one part of cells was stained by CD45, CD3, CD8, Granzyme B, CD4, CD25 and Foxp3 to evaluate changes in number of infiltrated CD8^+^T cells, Granzyme B^+^CD8^+^T cells and Foxp3^+^Tregs. All tumor samples were analyzed with flow cytometry and FlowJo software (Becton Dickinson).

### Statistical analysis

The software SPSS (version 20) and GraphPad Prism (version 7.0) were used for statistical analysis in our present study. Basically, difference among subgroups was analyzed by Student’s *t* test (2 subgroups) or one-way ANOVA (3 or more subgroups) method. And Spearman’s rank correlation analysis was used to assess the correlation between different items. Besides, difference in OS and RFS survival was calculated with Kaplan–Meier method, log-rank test and Hazard Ratio analysis. Data in TCGA were access online via access from the Kaplan-Meier Plotter website, with the best cutoff as the basis for classifying PDAC patients into high-expression and low-expression subgroups [50]. Gray values of the protein bands were calculated with Image J software and the relative expression level were labeled. The Bonferroni correction was used to correct for multiple testing when the *P* value needed correction. Overall, quantitative results were mean ± SD from at least 3 independent experiments, and *p*-value < 0.05 was considered significant in statistic.

## Supplementary information

Supplementary information

## Data Availability

The data that support the findings of this study are available from the corresponding author upon reasonable request.
