# Supplementary material for: Targeting MFAP5 in cancer-associated fibroblasts sensitizes pancreatic cancer to PD-L1-based immunochemotherapy via remodeling the matrix
Source: Oncogene. 2023 May 8;42(25):2061–73. doi: 10.1038/s41388-023-02711-9 (PMC10275759; doi:10.1038/s41388-023-02711-9)
Supplement: Supplementary file 1 — Supplementary information [file 41388_2023_2711_MOESM1_ESM.pdf]

# Supplementary Information

(Including Supplementary materials and methods, Figure S1-S10, Table S1-S4)

## Supplementary materials and methods

### Transfection, lentivirus infection and generation of stable transfected cells

Human PDAC CAFs (CAF3) and mouse PDAC CAFs (ImdyCAF) were used to construct stable MFAP5_NC or KD cell lines. The MFAP5 knockdown and negative control plasmids were ordered from the OBiO Technology. 293T cells at approximately 60% confluence were transiently co-transfected with the target plasmids and lentivirus package plasmids. HEK293T cells were cultured for 48h and the lentivirus supernatant was collected every 24h. The concentrated viral solution (with PEG8000) was further added to CAFs with proper concentration of polybrene (Sigma). Candidate CAFs were selected by puromycin (InvivoGen) and validated by western blot and qPCR assays after 24-48 hours' coculture. Construction of MFAP5_NC or OE CAFs was similar with the process described above, the control vector was appended with a RFP tag. In addition, transient transfection of siRNA targeting STAT1 was performed with lipo3000 (Invitrogen) according to the manufacturer's protocol.

### Cell proliferation and metastasis assay

Cell proliferation ability was assessed by Cell Counting Kit-8 (CCK-8, MCE), cell-light 5-Ethynyl-2'- deoxyuridine (EdU) staining kit (RIBOBIO) and flow cytometry analysis of cell cycle (PI staining; BD Pharmingen) according to the manufacturer's protocol. For analysis of metastasis capabilities, wound healing assay was performed

in low serum medium. Besides, migration and invasion (polycarbonate membrane were coated with Marigel (Corning, #354234) assays were performed in the 24-well transwell systems (8μm pores, Corning). Proper number ($5\text{-}10\times10^4$) of cells were seeded in the upper insets with serum-free medium whereas complete medium containing 20% FBS was added in the lower well. Cells that went through the membrane were observed and counted under microscope (Leica) after fixation and staining with crystal violet (Sigma-Aldrich).

**Three-dimensional (3D) spheroids formation assay, coculture and 3D culture system**

In the 3D spheroids cultivation system, hanging drop method was used to form spheroids containing MFAP5_KD or control CAFs, as described elsewhere.[16] CAFs were suspended to a proper concentration and cellular drops (20μl) were laid on the lid of culture plates. PBS was added to the plate for humidity and the lid was inverted allowing growth of the spheroids. Additionally, for 3D heterospheroids containing CAFs and PDAC cells, low-adhesion round-bottom 96-well plates were used and a 1:1 mixture of CAF subpopulations and KPC/Panc02 was seeded and incubated. Gemcitabine (10μM) and vehicle were added to the formed spheroids on the third day and refreshed on the sixth day. For both spheroid systems, morphology and diameter of spheroids was observed and imaged under a microscope. Viability of spheroids were detected with CellTiter-Lumi™ Steady Plus Kit (Beyotime) following the manufacturer's protocol. As for the coculture system, conditioned medium from MFAP5_NC or KD CAFs (collected from equal amount for equal duration) were added

to the candidate cells and the conditioned medium was refreshed every 24 hours. Additionally, the 3D tube formation assay was performed in 96-well plate. 50ul Matrigel was lined at the bottom of the board and wait 30 minutes until solidification, then $2\times10^3$ HUVEC were seeded in 100ul Matrigel (diluted by DMEM culture medium, 1:1). Stretch of tubes were then examined under a microscope in 1-3 days and the images were then analyzed by Image J 1.8.0 software.

**Nuclear-cytoplasmic separation, immunoprecipitation and Western blot assay**

The separation of nuclear and cytoplasmic protein was carried out with Nuclear and Cytoplasmic Extraction Reagent (ThermoFisher SCIENTIFIC) according to the manufacturer's protocol. Immunoprecipitation and western blot assay were performed as previously described.[40] Information of antibody involved in the present study was listed in Table S3. The protein signals were detected and visualized with the ChemiScopeTouch (Clinx Science Instruments, Shanghai, China), and the intensity of western blot bands was measured by Image J 1.8.0 software.

**RNA isolation, qRT-PCR, dual-luciferase reporter assay and chromatin immunoprecipitation (ChIP) assay**

Cellular total RNA was extracted with TRIzol reagents (Invitrogen) and qRT-PCR was performed according to standard protocols as previously described.[40] Chromatin immunoprecipitation (ChIP) assay was performed with ChIP Assay Kit (Beyotime) according to the manufacturer's protocols. The promoter regions containing 3000bp upstream sequence as well as 5’UTR were cloned into the luciferase reporter vector.

Two most predicted binding sites were mutant defined as mut1 and mut2. Relative luciferase activity was measured with dual-luciferase reporter assay system (Promega) according to the manufacturer's protocols. Primer sequences were listed in Table S4.

**IHC, chemical staining and Immunofluorescence staining**

Immunohistochemistry (IHC) and immunofluorescence staining were performed as previously described (38). In our present study, staining of stromal deposited collagen was performed with Picrosirius Red Stain Kit (Polysciences, INC) and hyaluronic acid staining was performed with Alcian blue - Nuclear solid red staining Kit (Beyotime) following the manufacturer's protocols. Besides, IHC staining of HAS2 was also performed considering the non-specific staining of both collagen and mucins with Alcian blue alone. Notably, most of our findings were verified with more than one method in at least 3 repeated individual experiments, and optimal statistical methods were performed to evaluate the differences. The multiplex immunofluorescence was performed according to the manufacture's protocol via usage of Akoya Biosciences Polaris Kit.

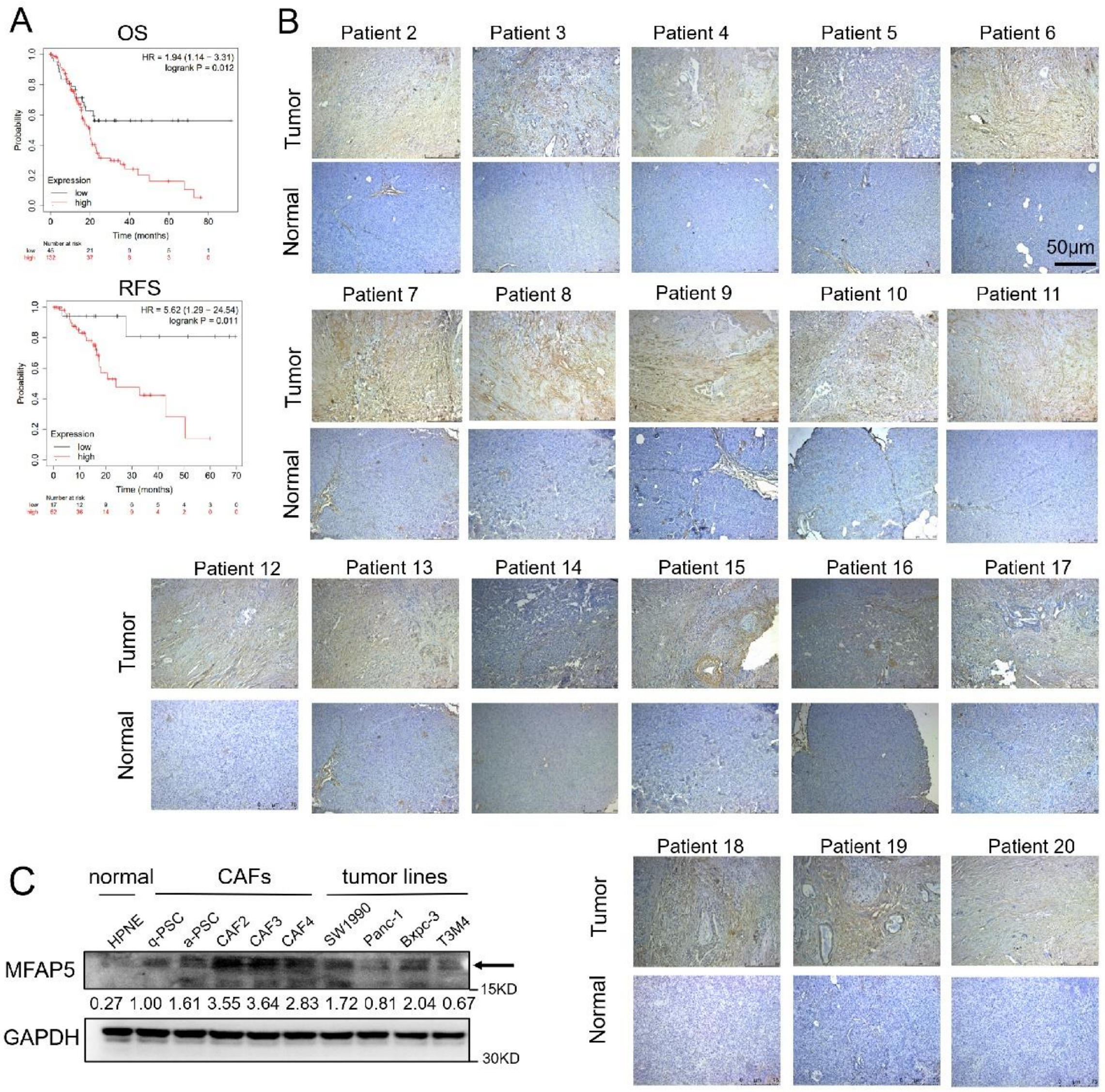


**Figure S1 Additional results of MFAP5 expression in PDAC.** (A) Kaplan-Meier OS and RFS survival curves for overall mRNA expression of MFAP5 in patients with PDAC in TCGA database. The best cutoff was used to classify the patients into high and low expression subgroups.(B) Additional images of MFAP5 IHC staining in 19 paired human tumor (T) and adjacent (N) pancreatic tissues. Scale bars, 50μm. (C) Western-blot analysis of MFAP5 in different pancreatic cell lines.

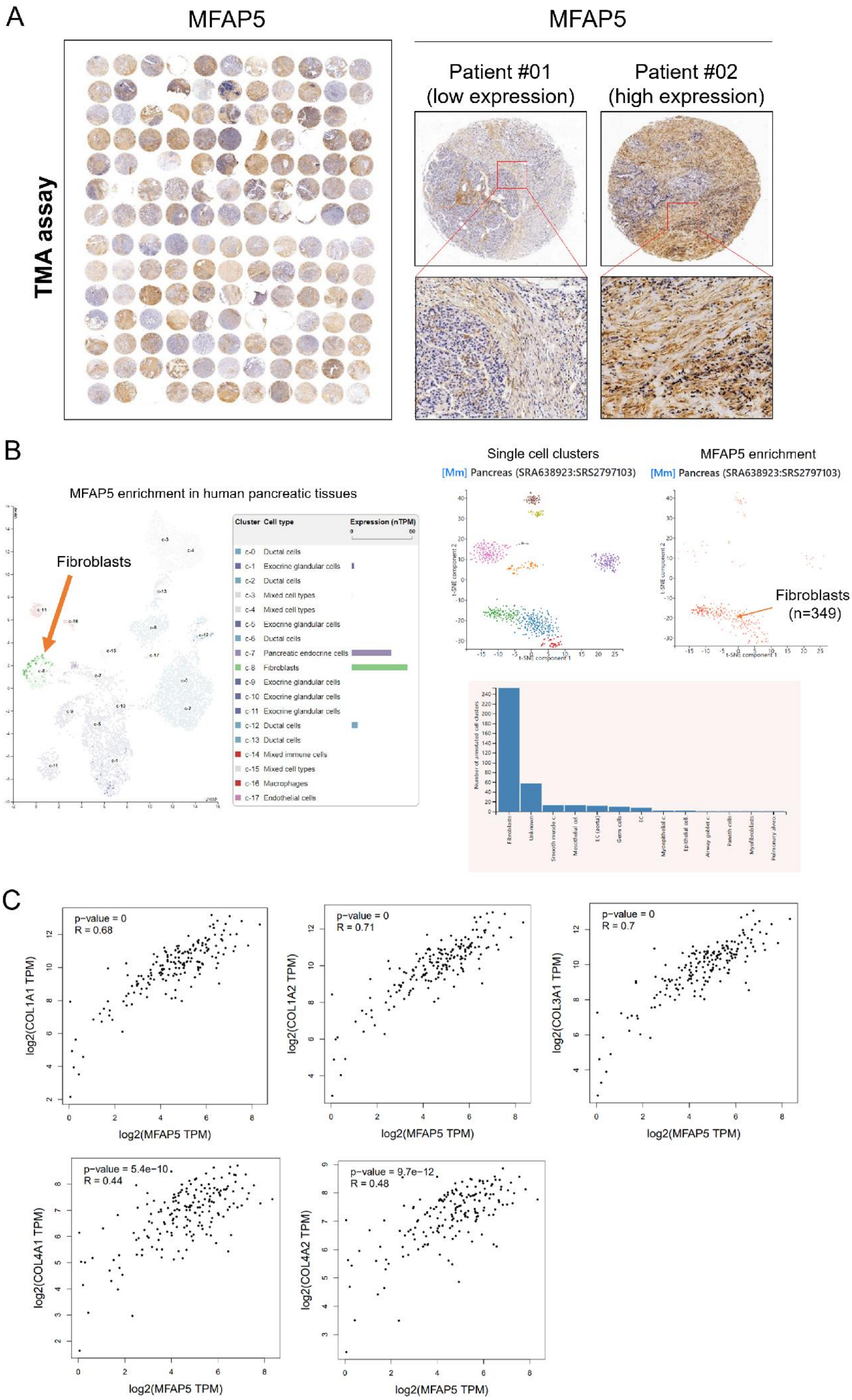
A
MFAP5
MFAP5
TMA assay
Patient #01
(low expression)
Patient #02
(high expression)
B
MFAP5 enrichment in human pancreatic tissues
Fibroblasts
Cluster Cell type
Expression (nTPM)
c-0 Ductal cells
c-1 Exocrine glandular cells
c-2 Ductal cells
c-3 Mixed cell types
c-4 Mixed cell types
c-5 Exocrine glandular cells
c-6 Ductal cells
c-7 Pancreatic endocrine cells
c-8 Fibroblasts
c-9 Exocrine glandular cells
c-10 Exocrine glandular cells
c-11 Exocrine glandular cells
c-12 Ductal cells
c-13 Ductal cells
c-14 Mixed immune cells
c-15 Mixed cell types
c-16 Macrophages
c-17 Endothelial cells
Single cell clusters
[Mm] Pancreas (SRA638923:SRS2797103)
MFAP5 enrichment
[Mm] Pancreas (SRA638923:SRS2797103)
Fibroblasts
(n=349)
t-SNE component 1
t-SNE component 2
Number of annotated cell clusters
Fibroblasts
Unknown
EC (aorta)
Germ cells
EC
Epithelial cell
Paneth cells
Myofibroblasts
C
p-value = 0
R = 0.68
log2(COL1A1 TPM)
log2(MFAP5 TPM)
p-value = 0
R = 0.71
log2(COL1A2 TPM)
log2(MFAP5 TPM)
p-value = 0
R = 0.7
log2(COL3A1 TPM)
log2(MFAP5 TPM)
p-value = 5.4e-10
R = 0.44
log2(COL4A1 TPM)
log2(MFAP5 TPM)
p-value = 9.7e-12
R = 0.48
log2(COL4A2 TPM)
log2(MFAP5 TPM)

**Figure S2 Additional information of roles of MFAP5 in PDAC.** (A) Tumor tissue arrays (TMA) assay and representative images of MFAP5 staining. (B) Single cell analysis of MFAP5 in PDAC tissues from The Human Protein Atlas database. Density of colored points represents the relative expression according to fixed interval of read count (0, 1, 2-4, 5-9, >10). Column diagram is shown on the right. MFAP5 enrichment in single cell sequencing clusters of mouse pancreas tissues in PanglaoDB database. (C) Gene co-expression analysis between MFAP5 and collagen-related genes.

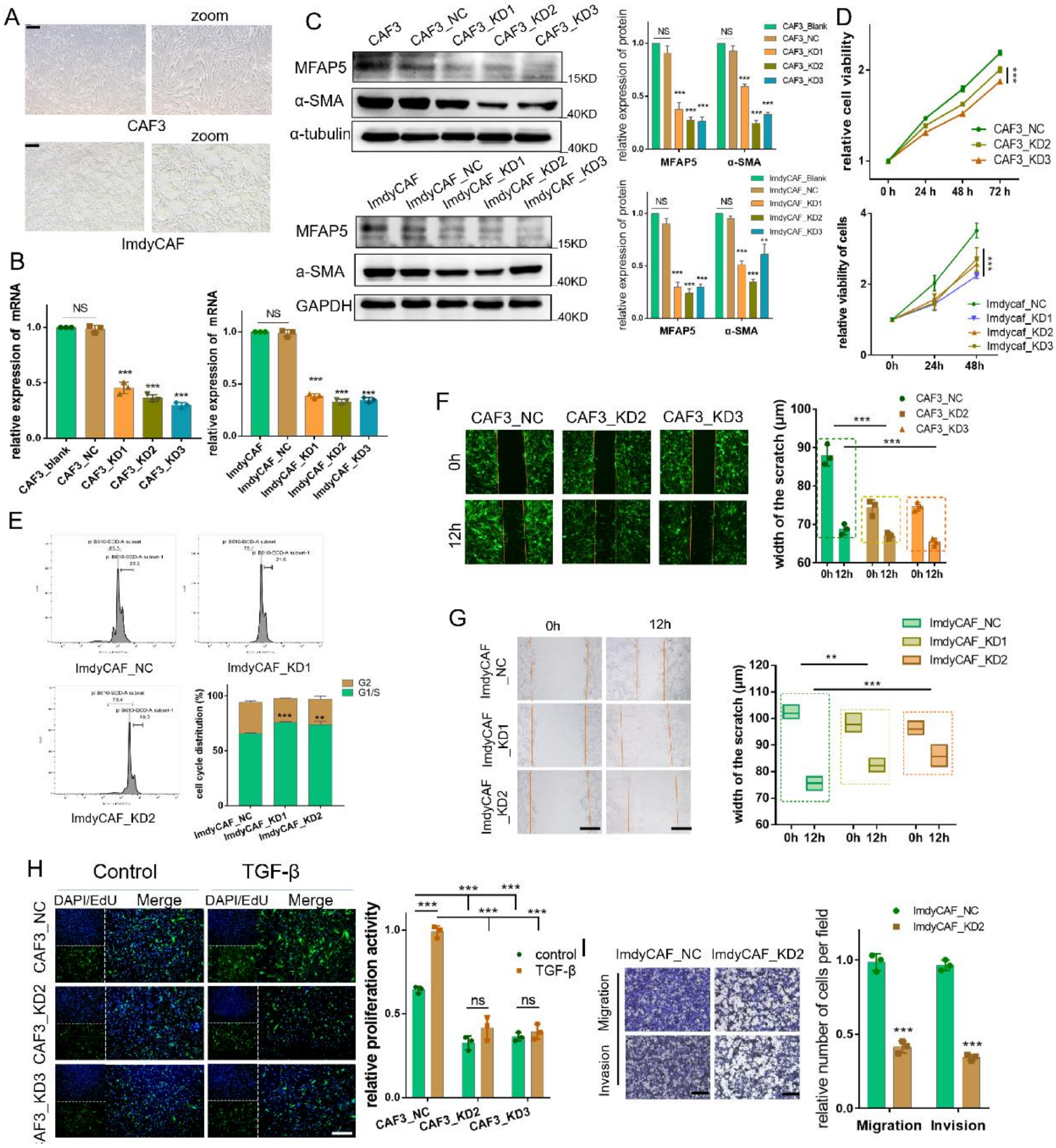

**Figure S3 Additional results of MFAP5 knockdown in CAFs.** (A) Representative images of morphological characteristics of CAFs. CAF3 is are derived from human PDAC tissues and ImdyCAF is derived from mouse PDAC tissues. Scale bar: 100μm. (B and C) Verification of knockdown efficiency in stable transfected CAFs in mRNA(B) and protein(C) levels. (D and E) Relative cell viability of MFAP5_KD or control CAFs with CCK-8 assay and cell cycle analysis. (F-G) Wound healing assay in MFAP5_KD CAFs/ImdyCAFs compared with control CAFs/ImdyCAFs cultured in low-serum medium. Scale bar: 50μm. (H) Cell proliferation of MFAP5_KD or control CAFs with or without TGF-β assessed by EdU assay. Scale bar: 50μm (I) Trans-well experiment indicates migration and invasive capabilities of both MFAP5_KD or control /ImdyCAFs. The data were analyzed by a two-tailed unpaired Student's t-test (B, C, D, E, F, G, H, I). Error bars, means ± SD of three independent experiments. *P < 0.05, **P < 0.01, *** P < 0.001, ns, not significant.

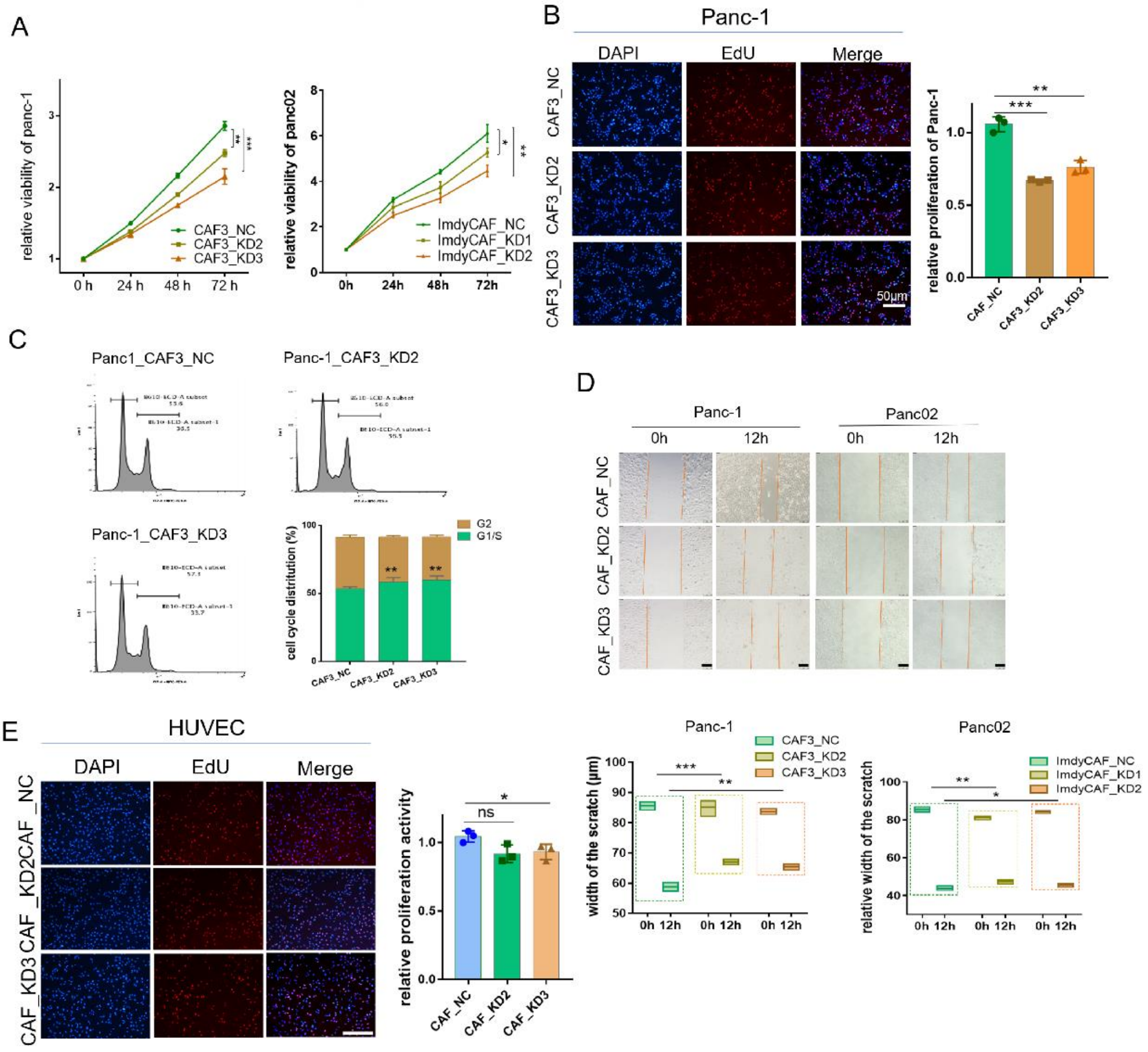


**Figure S4 Additional results of influence of MFAP5 knockdown on tumor cells.** (A) Relative growth rate of Panc-1 and Panc02 cocultured with homologous stable-transfected CAFs with CCK-8 assay. Cells were cultured in the conditioned medium (refreshed every day) throughout the assays. (B) Relative growth rate of Panc-1 cocultured with homologous stable-transfected CAFs with EdU assay. Scale bar: 50μm. (C) Cell cycle analysis of Panc-1 in cocultured systems with flow cytometry. (D) Wound healing assay of Panc-1 and Panc02 in cocultured system with low-serum medium. Scale bar: 100μm. (E) Representative images of cell viability of HUVEC in cocultured system with EdU assay. Scale bars, 50μm. The data were analyzed by a two-tailed unpaired Student's t-test (A, B, C, D, E). Error bars, means ± SD of three independent experiments. *P < 0.05, **P < 0.01, *** P < 0.001, ns, not significant.

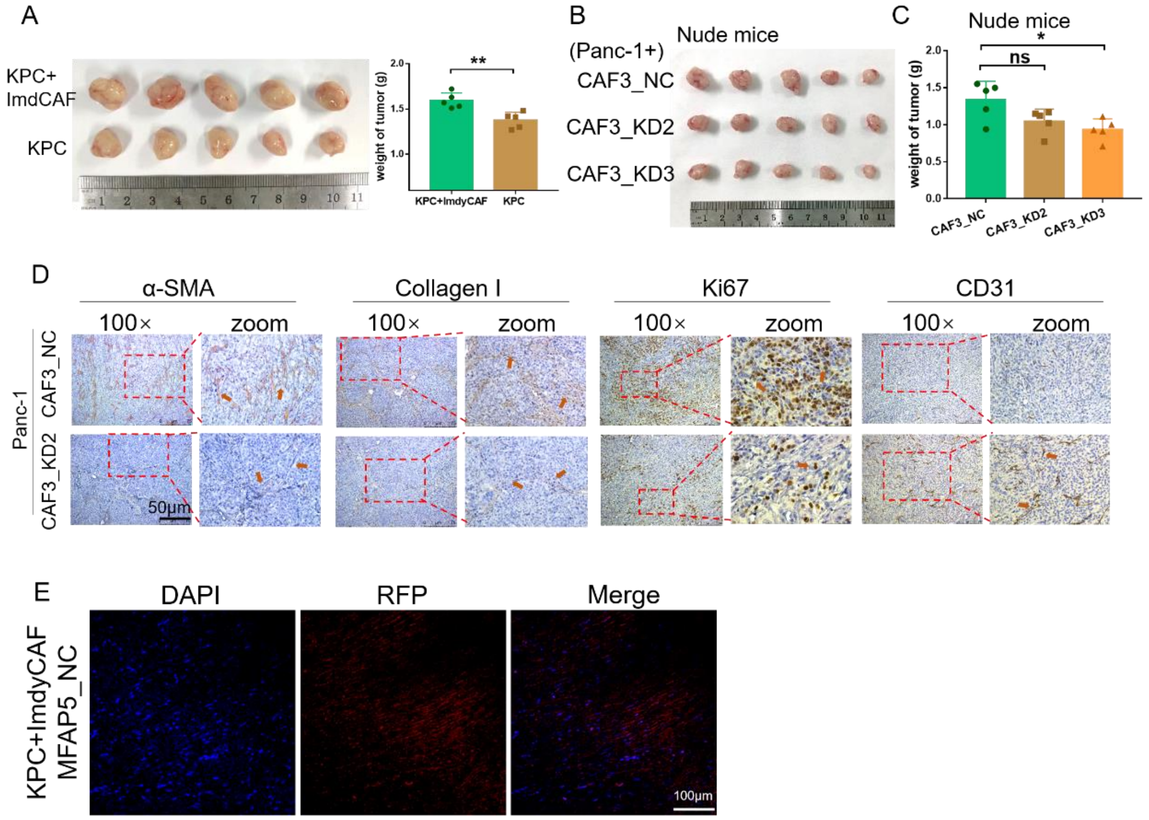


**Figure S5 Additional results of roles of MFAP5 *in vivo*.** (A) Images and statistical weights of tumors derived from KPC or mixture co-injection of KPC and ImdyCAF in C67BL/6 mice. (B and C) Images and statistical tumor weights of isolated co-injected KPC tumors derived from nude mice. (D) Additional representative IHC staining of α-SMA, Collagen I, Ki67 and CD31 in tumors from nude mice in Figure 3B. Scale bars, 50μm, 100μm, 200μm. (E) Representative images of immunofluorescent staining of RFP in C67BL/6 mice. The ImdyCAFs were stably transfected with vector with RFP tag. The data were analyzed by a two-tailed unpaired Student's t-test (A, B). Error bars, means ± SD of three independent experiments. $^{**}P < 0.01$, ns, not significant.

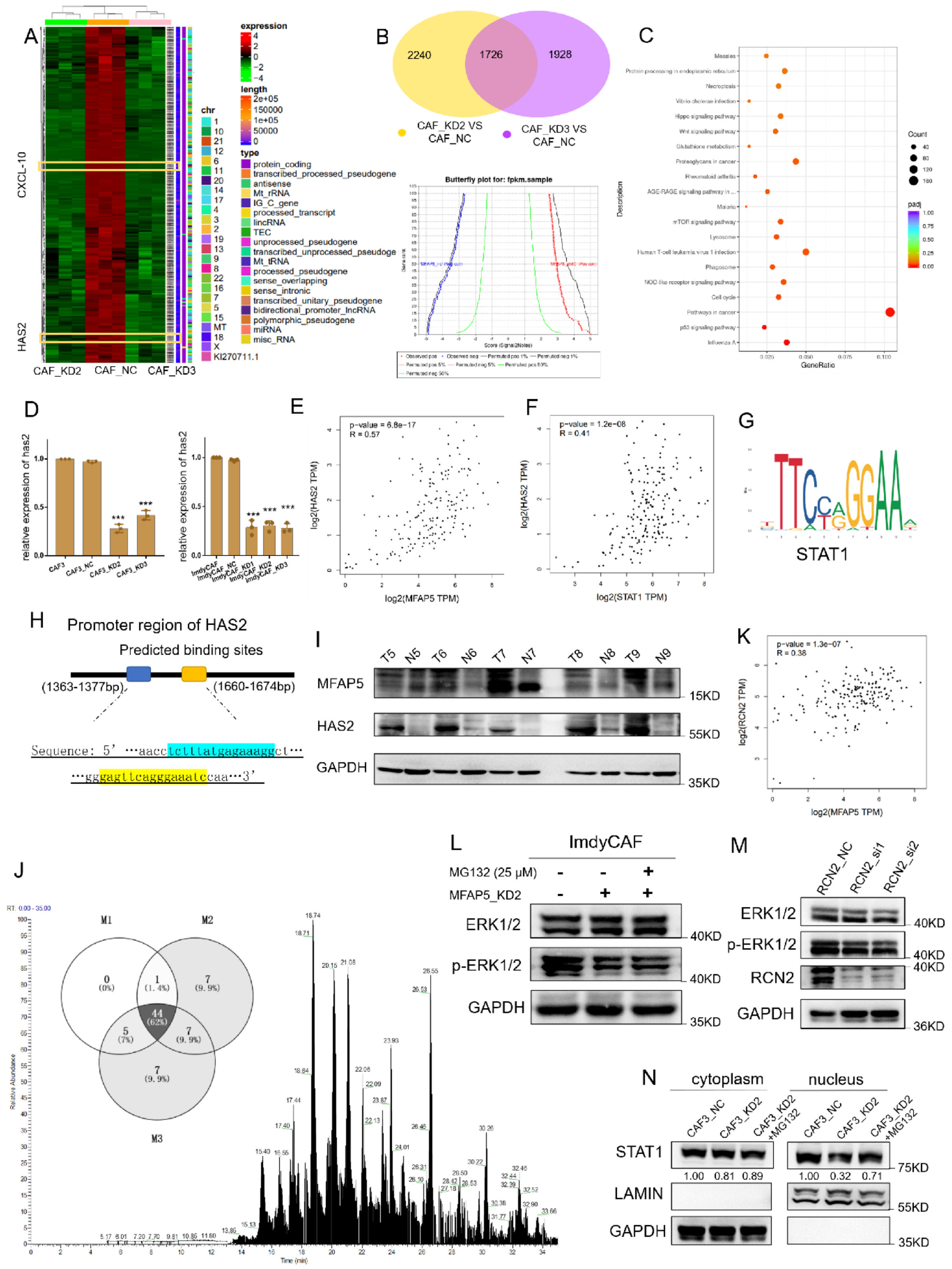


**Figure S6 Additional results of MFAP5 promotes transcription of HAS2.** (A) Differential genes of MFAP5_KD or control CAFs in heat map with indicated downregulation of HAS2 and CXCL10 when MFAP5 is deficient. (B) Venn diagram and butterfly diagram for downstream gene screening and validation. (C) KEGG analysis results in bubble diagram. Pathway items are shown on the left. P-value is indicated with color and item counts are

indicated with different size of bubbles. (D) Validation of HAS2 mRNA expression in MFAP5_KD or control CAFs via PCR experiment. (E, F and K) Gene co-expression analysis of HAS2 and MFAP5(E), STAT1 and HAS2(F), RCN2 and MFAP5 (K). (G and H) Representative images indicating binding sites of STAT1(G) on promoter regions of HAS2(H) (two most significant sites) predicted in the JASPAR database. The predicted sequences were highlighted. (I) Western blot analysis of MFAP5 and HAS2 in paired tumor or adjacent pancreas tissues. (J) Representative ion profile of proteome analysis and venn diagram of different subgroups. (L) Western blot of ERK/p-ERK in MFAP5_KD or control CAFs with or without MG132 (25μM, 24h). (M) Western blot assay indicating changes in ERK pathways when RCN2 was interfered with siRNA. (N) Western blot assay showing roles of MG132 on the decreased STAT1 due to deficient MFAP5. The data were analyzed by a two-tailed unpaired Student's t-test (D). Error bars, means ± SD of three independent experiments. *** $P < 0.001$.

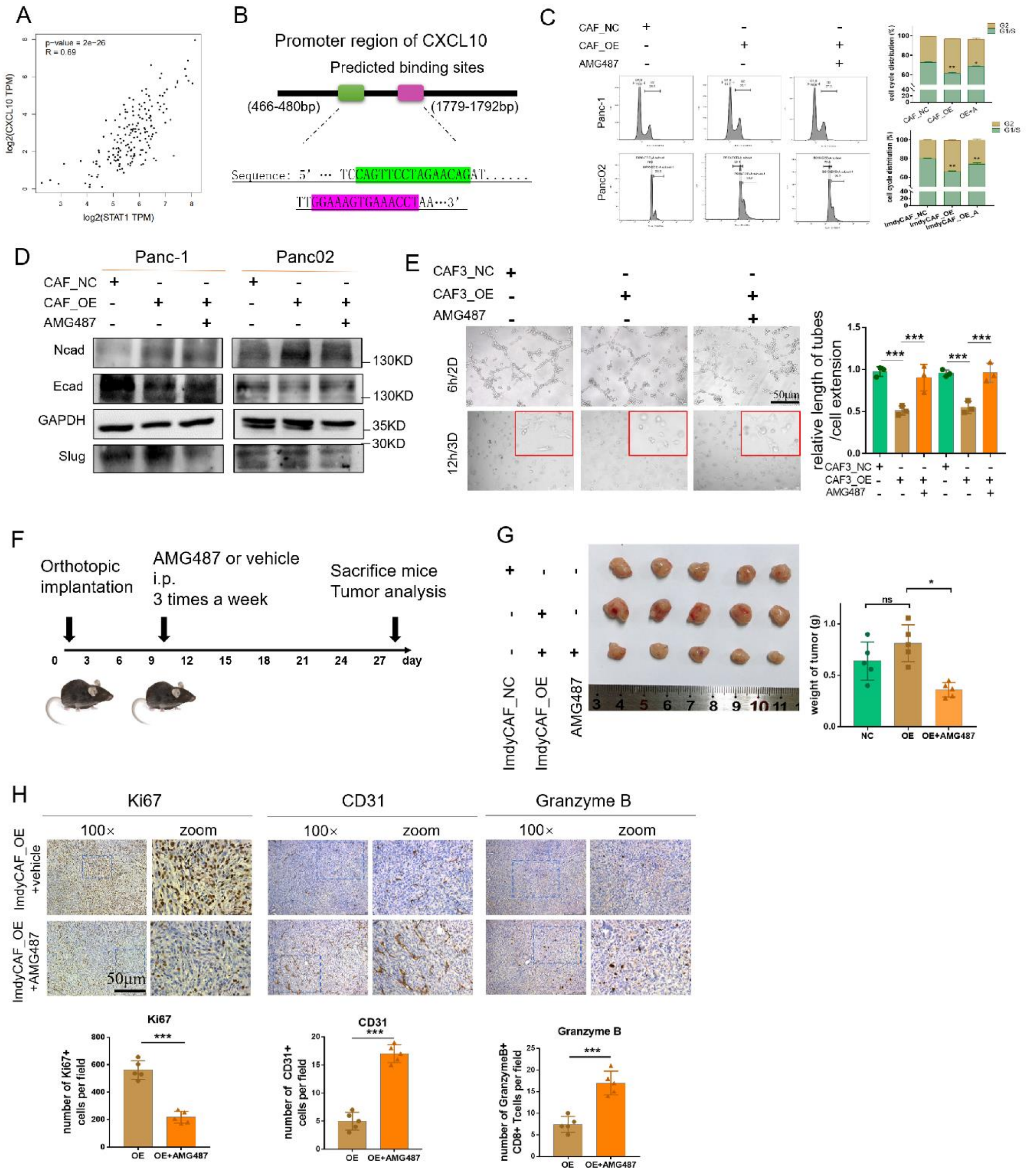


**Figure S7 Blockade of CXCL10 is in synergy with anti-PD-L1 treatment.** (A) Gene co-expression analysis of STAT1 and CXCL10 (R=0.69, p-value=2e-26) and representative images indicating binding sites of STAT1 on promoter regions of CXCL10 (two most significant sites) predicted in the JASPAR database. The predicted sequences were highlighted. (B) Secretion of CXCL12 and IL-8 when MFAP5 was deficient in CAF3 via ELISA assay. (C) Cell cycle analysis of Panc-1 and Panc02 cells in the cocultured systems treated with AMG487 (5nm, 24h). (D) Western analysis of EMT-related proteins of Panc-1 and Panc02 cells in coculture system with or without AMG487 (5nM, 24h). (E) Representative images of 2D/3D tube formation assays of HUVEC cells cocultured in coculture system with or without AMG487(5nM, 24h). Scale bar: 50μm. (F) Schematic procedures of orthotopic co-injection of ImdyCAFs and KPC cells into C57BL/6 mice combined with administration of AMG487 (5mg/kg). (G) Images and

statistical tumor weight of isolated tumors derived from Panc02 and MFAPF_OE or control ImdyCAFs with administration of AMG487 (5mg/kg). A total of 15 mice were analyzed (5 mice for each group individually). (H) Representative IHC staining images and histograms of tumors in Figure 5J, and the statistical analysis of Ki67, CD31 and Granzyme B. (I) Roles of AMG487 on tumors generated from KPC cell lines alone. Statistical results of the tumor weight and survival benefit were shown on the right. Scale bar: 50μm. The data were analyzed by a two-tailed unpaired Student's t-test (C, E, G, H, I). Error bars, means ± SD of three independent experiments. *P < 0.05, **P < 0.01, *** P < 0.001, ns, not significant.

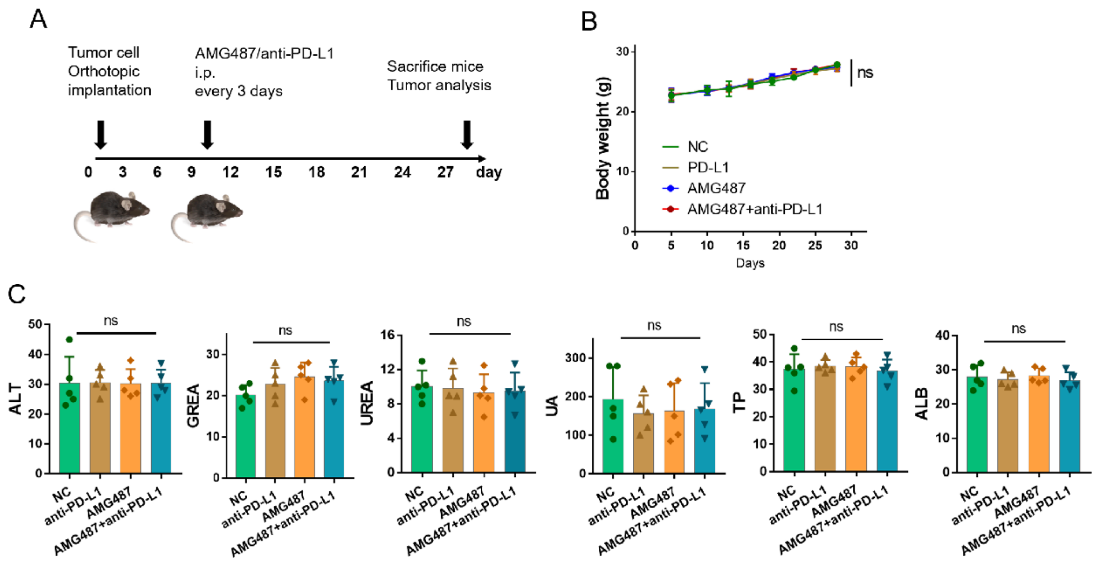


**Figure S8 Additional results of MFAP5 regulates tumoral PD-L1 via CXCL10.** (A) Schematic procedures of orthotopic co-injection of ImdyCAFs and KPC cells into C57BL/6 mice combined with administration of AMG487 (5mg/kg) and anti-PD-L1 antibody (200μg/mouse). (B) Changes of mouse body weight during the experiment. (C) Detection of serum chemical indicators of mice cotreated with AMG487 (5mg/kg) and anti-PD-L1 antibody. Error bars, means ± SD of three independent experiments. *P < 0.05, **P < 0.01, *** P < 0.001, ns, not significant.

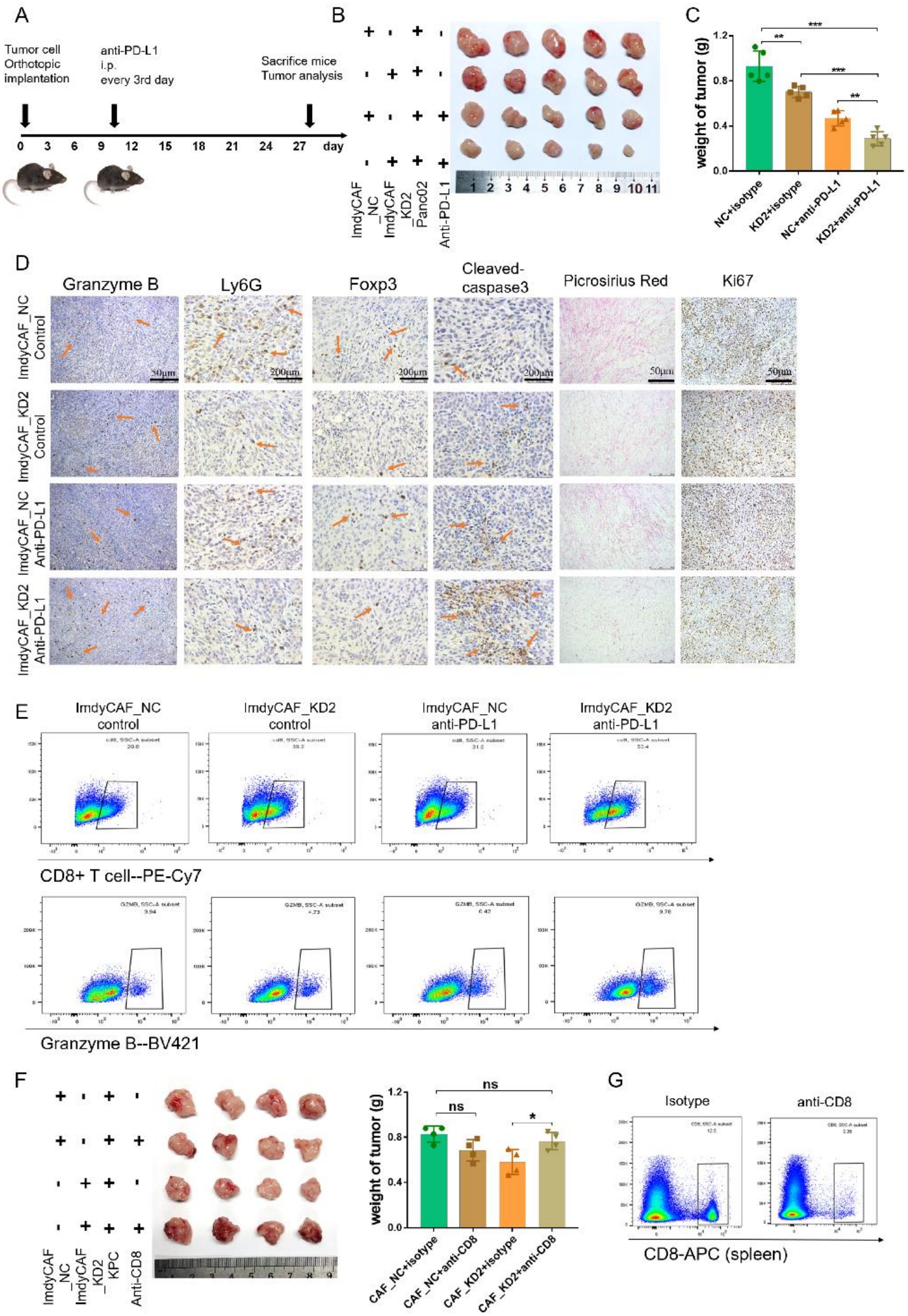


**Figure S9 Additional results of MFAP5 inhibition in CAFs synergizes PD-L1 based immunotherapy.** (A) Schematic procedures of orthotopic co-injection of stable transfected

ImdyCAFs and KPC or Panc02 cells in C57BL/6 mice combined with administration of anti-PD-L1 antibody (200μg/mouse). (B-C) Images and statistical tumor weights of isolated Panc02 tumors co-injected with MFAP5_KD or control CAFs administrated with/without anti-PD-L1 antibody. (D) Representative images and statistical analysis of IHC staining of Granzyme B, CD31, Foxp3, Cleaved-caspase3 and chemical staining of collagen deposition (picrosirius red) of tumors in Figure 6F. Scale bars, 50μm (E) Flow cytometry of infiltrated $CD8^{+}$T cells, Granzyme $B^{+}CD8^{+}$T cells. (F). CD8 deletion assay *in vivo* with anti-CD8 antibody (Bio X cell). (G) Validation of CD8 deletion effect by flow cytometry analysis of single cells of spleen. The data were analyzed by a two-tailed unpaired Student's t-test (B, F). Error bars, means ± SD of three independent experiments. *$P < 0.05$, **$P < 0.01$, *** $P < 0.001$, ns, not significant.

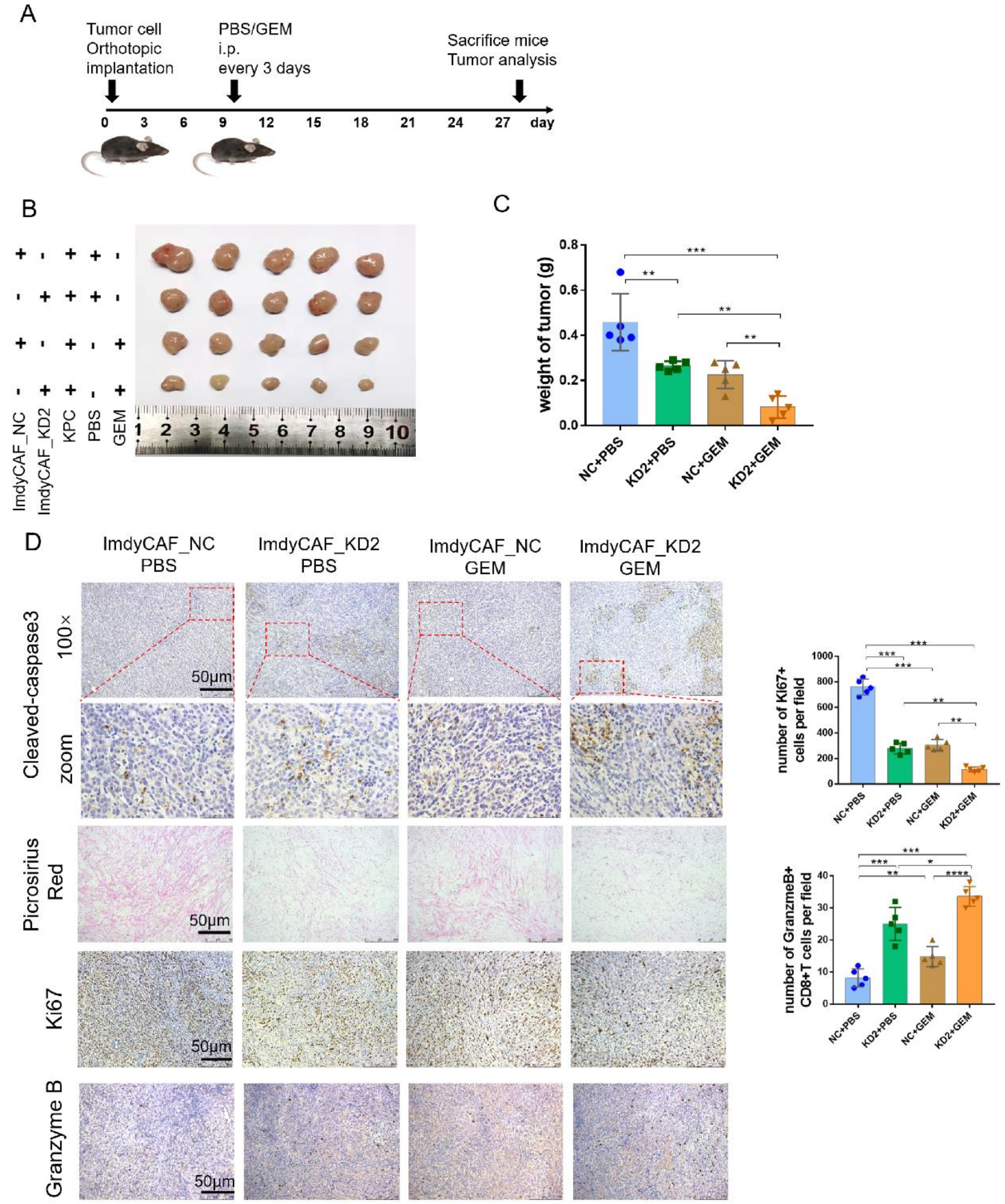


**Figure S10 Additional results of MFAP5 inhibition in CAFs synergizes gemcitabine.** (A) Schematic procedures of orthotopic co-injection of stable transfected ImdyCAFs and Panc02 or KPC cells into C57BL/6 mice combined with administration of gemcitabine (50mg/kg) every three days. (B) Images and (C) statistical tumor weight of isolated co-injected KPC tumors after mice were sacrificed at day 28. A total of 20 mice were analyzed (5 mice for each group individually). (D) Representative IHC staining images and statistical analysis of Ki67 and Granzyme B. Scale bar: 50μm. The data were analyzed by a two-tailed unpaired Student's t-test (C, D). Error bars, means ± SD of three independent experiments. *P < 0.05, **P < 0.01, *** P < 0.001.

**Table S1 Association between MFAP5 and the corresponding clinicopathological information of PDAC patients**

| Variables | MFAP5 expression in tissue | | *P* value |
|---|---|---|---|
| | Low level (H-score≤90, n=70) | High level (H-score>90, n=70) | |
| **Gender** | | | |
| Male | 39(55.71%) | 42(60.00%) | 0.7323 |
| Female | 31(44.29%) | 28(40.00%) | |
| **Age(years)** | | | |
| >60 | 58(82.86%) | 61(87.14%) | 0.6368 |
| ≤60 | 12(17.14%) | 9(12.86%) | |
| **TNM stage** | | | |
| I-II stage | 55(78.57%) | 43(61.43%) | **0.0226*** |
| III-IV stage | 13(18.57%) | 26(37.14%) | |
| Missing | 2(2.86%) | 1(1.43%) | |
| **Vascular invasion** | | | |
| Yes 71 | 33(47.14%) | 39(55.71%) | 0.3943 |
| No 80 | 36(51.43%) | 30(42.86%) | |
| Missing | 1(1.43%) | 1(1.43%) | |
| **Nerve invasion** | | | |
| Yes | 40(57.14%) | 54(77.14%) | **0.0171*** |
| No | 29(41.43%) | 15(21.43%) | |
| Missing | 1(1.43%) | 1(1.43%) | |
| **CA12-5(U/mL)** | | | |
| ≥35 | 19(27.14%) | 13(18.57%) | 0.2189 |
| <35 | 42(60.00%) | 51(72.865%) | |
| Missing | 9(12.86%) | 6(8.57%) | |
| **CA19-9(U/mL)** | | | |
| ≥37 | 51(72.86%) | 60(85.71%) | 0.0546 |
| <37 | 18(25.71%) | 9(12.86%) | |
| Missing | 1(1.43%) | 1(1.43%) | |
| **CEA(U/mL)** | | | |
| ≥5 | 17(24.29%) | 25(35.71%) | 0.1339 |
| <5 | 47(67.14%) | 38(54.29%) | |
| Missing | 6(8.57%) | 7(10.00%) | |
| **Tumor differentiation** | | | |
| Moderate - Well | 52(74.29%) | 36(51.43%) | **0.0431*** |
| Poor | 16(22.86%) | 30(42.86%) | |
| Missing | 2(2.86%) | 4(5.71%) | |
| **Recurrence** | | | 0.3703 |
| Yes | 31 (44.29%) | 38(54.29%) | |

| | | |
|---|---|---|
| No | 16(22.86%) | 12(17.14%) |
| Missing | 23(32.86%) | 20(28.57%) |

*Indicates *p*-value < 0.05. Analyzed with Fisher's exact test.

**Table S2 Association between serous MFAP5 and the corresponding clinicopathological information in PDAC patients.**

| Variable | MFAP5 expression in serum | | *P* value |
|---|---|---|---|
| | Low level (≤4.25 ng/ml, n=23) | High level (≥4.25 ng/ml, n=27) | |
| **Gender** | | | |
| Male | 15(65.22%) | 13(48.15%) | 0.2641 |
| Female | 8(34.78%) | 14(51.85%) | |
| **Age(years)** | | | |
| >60 | 20(86.96%) | 20(77.07%) | 0.3079 |
| ≤60 | 3(13.04%) | 7(25.93%) | |
| **TNM stage** | | | |
| I-II stage | 21(91.30%) | 21(77.78%) | 0.2609 |
| III-IV stage | 2(8.70%) | 6(22.22%) | |
| **Vascular invasion** | | | |
| Yes | 10(40.00%) | 17(62.96%) | 0.1644 |
| No | 15(60.00%) | 10(37.04%) | |
| **Nerve invasion** | | | |
| Yes | 20(86.96%) | 18(66.67%) | 0.1119 |
| No | 3(13.04%) | 9(33.33%) | |
| **CA12-5(U/mL)** | | | |
| ≥35 | 4(17.39%) | 10(37.04%) | 0.2060 |
| <35 | 19(82.61%) | 17(62.96%) | |
| **CA19-9(U/mL)** | | | |
| ≥37 | 17(73.91%) | 25(92.59%) | 0.1214 |
| <37 | 6(26.09%) | 2(7.41%) | |
| **CEA(U/mL)** | | | |
| ≥5 | 5(21.74%) | 16(59.29%) | **0.0102*** |
| <5 | 18(78.26%) | 11(40.74%) | |
| **Tumor differentiation** | | | |
| Moderate – Well | 19(82.61%) | 14(51.85%) | **0.0355*** |
| Poor | 4(17.39%) | 13((48.15%) | |
| **Recurrence** | | | |
| Yes | 11(47.83%) | 19(70.37%) | 0.1495 |
| No | 12(52.17%) | 8(29.63%) | |

* Indicates *p*-value < 0.05. Analyzed with Fisher's exact test.

**Table S3 Antibodies used in the study**

| Antibody | Catalog number | Manufacture |
|---|---|---|
| Asma | ab7817 | Abcam |
| CollagenI | 14695-1-AP | Proteintech |
| FAP | ab207178 | Abcam |
| CK19 | 12434 | Cell Signaling Technology |
| RCN2 | 10193-2-AP | Proteintech |
| STAT1 | 14994T | Cell Signaling Technology |
| Anti-STAT1 - ChIP Grade | ab234400 | Abcam |
| pSTAT1 | ab109461 | Abcam |
| pSTAT1 | 7649T | Cell Signaling Technology |
| NF-KB(p65) | ab218533 | Abcam |
| LAMIN | 4777 | Cell Signaling Technology |
| Epithelial-Mesenchymal Transition (EMT) Antibody Sampler Kit | 9782T | Cell Signaling Technology |
| pAKT | 4060 | Cell Signaling Technology |
| MTOR | ab109268 | Abcam |
| KI67 | 9449 | Cell Signaling Technology |
| CD31 | 3528 | Cell Signaling Technology |
| Cleaved Caspase-3 | 9664 | Cell Signaling Technology |
| LY6G | 87048 | Cell Signaling Technology |
| GAPDH | 100ul | Beyotime |
| sc-365263 | HAS2 (C-5) | Santa Cruz |
| FoxP3 | 12653 | Cell Signaling Technology |
| MFAP5 | ab232846 | Abcam |
| anti-PD-L1 | 13684 | Cell Signaling Technology |
| anti-PD-L1 | ab213480 | Abcam |
| anti-PD-L1 | ab205921 | Abcam |
| anti-Ubiquitin | 3933 | Cell Signaling Technology |
| anti-CD8a | 98941 | Cell Signaling Technology |
| anti-GAPDH | AF5009 | Beyotime |
| anti-α-tubulin | AF0001 | Beyotime |
| anti-HA-Tag | 3724 | Cell Signaling Technology |
| rabbit IgG isotype control | 8726 | Cell Signaling Technology |
| mouse IgG isotype control | sc-2025 | Santa Cruz |
| HRP goat anti-rabbit IgG | A0208 | Beyotime |
| HRP goat anti-mouse IgG | A0216 | Beyotime |
| goat anti-rabbit IgG | GTX77061 | GeneTex |
| goat anti-mouse IgG | GTX26708 | GeneTex |
| FITC anti-mouse CD3 | 100203 | Biolegend |
| APC/Cy7 anti-mouse CD3 | 100329 | Biolegend |
| Brilliant Violet 785 anti-mouse CD45 | 304048 | Biolegend |

| | | |
|---|---|---|
| PE/Cy7 anti-mouse CD8a | 100722 | Biolegend |
| Brilliant Violet 605 anti-mouse CD8a | 100744 | Biolegend |
| Brilliant Violet 421 anti-human/mouse Granzyme B Recombinant | 396414 | Biolegend |
| BV785 anti-human CD45 | 304047 | Biolegend |
| PE anti-mouse CD274 | 124308 | Biolegend |
| Brilliant Violet 421 anti-human CD274 | 329714 | Biolegend |
| PE rat IgG2b κ isotype control | 400607 | Biolegend |
| Brilliant Violet 421 mouse IgG2b κ isotype control | 400342 | Biolegend |
| PE rat anti-mouse IgG1 | 550083 | BD biosciences |
| InVivoMAb anti-mouse PD-L1 | BE0101 | BioXcell |
| IgG isotype control | BE0090 | BioXcell |
| PE anti-human CXCL10 (IP-10) Antibody | 519503 | Biolegend |
| PE/Cyanine7 anti-human IL-8 Antibody | 511415 | Biolegend |

**Table S4 Primer sequence**

| gene _name | sequence | |
|---|---|---|
| h_MFAP5 | CCTGGGGGTCAATAGTCAACGA | CTCATCCCAGCACTCCAAGTC |
| m_MFAP5 | ACCTTCCACAGATGACCTAGC | TCACAGGGAGGAAGTCGGAA |
| RCN2 | GCAAGAAGCCAAGCAGCAGTTTG | GATCCCGCCTGTAATCGCCAAG |
| STAT1 | ACCGCACCTTCAGTCTTTTC | CTCATTCACATCTCTCAACTTCACA |
| h_HAS2 | GTCGAGTTTACTTCCCGCCA | TTCCGCCTGCCACACTTATT |
| m_HAS2 | GCCTCGCATCTCATCATCCA | CCTGCCCATAACTTCGCTGA |
| h_CXCL10 | TGCCATTCTGATTTGCTGCCT | TGATGCAGGTACAGCGTACAG |
| m_CXCL10 | CAAGTGCTGCCGTCATTTTCT | AGGATAGGCTCGCAGGGATG |
| SPARC | CCCTGGCAGCCCCTCA | CTCTCGTCCAGCTCACACAC |
| COL1A1 | GCAAGAGGCGAGAGAGGTTT | GACCACGGGCACCATCTTTA |
| Acta2 (α-SMA) | ACTGGGACGACATGGAAAAG | GTTCAGTGGTGCCTCTGTCA |
| HGF | TTGGGATTCGCAGTACCCTCACAA | TAGCCAACTCGGATGTTTGGGTCA |
